# Mapping Neural Networks to FPGA-Based IoT Devices for Ultra-Low Latency Processing

**DOI:** 10.3390/s19132981

**Published:** 2019-07-05

**Authors:** Maciej Wielgosz, Michał Karwatowski

**Affiliations:** 1 Faculty of Computer Science, Electronics and Telecommunications, AGH University of Science and Technology, al. Adama Mickiewicza 30, 30-059 Cracow, Poland; 2 Academic Computer Centre CYFRONET AGH, ul. Nawojki 11, 30-072 Cracow, Poland

**Keywords:** neural networks, internet of things (IoT), FPGA, deep learning, recurrent neural network (RNN)

## Abstract

Internet of things (IoT) infrastructure, fast access to knowledge becomes critical. In some application domains, such as robotics, autonomous driving, predictive maintenance, and anomaly detection, the response time of the system is more critical to ensure Quality of Service than the quality of the answer. In this paper, we propose a methodology, a set of predefined steps to be taken in order to map the models to hardware, especially field programmable gate arrays (FPGAs), with the main focus on latency reduction. Multi-objective covariance matrix adaptation evolution strategy (MO-CMA-ES) was employed along with custom scores for sparsity, bit-width of the representation and quality of the model. Furthermore, we created a framework which enables mapping of neural models to FPGAs. The proposed solution is validated using three case studies and Xilinx Zynq UltraScale+ MPSoC 285 XCZU15EG as a platform. The results show a compression ratio for quantization and pruning in different scenarios with and without retraining procedures. Using our publicly available framework, we achieved 210 ns of latency for a single processing step for a model composed of two long short-term memory (LSTM) and a single dense layer.

## 1. Introduction

Artificial Intelligence algorithms are developing rapidly and in multiple areas. One of the crucial factors causing this significant leap is the exponential increase of the available data, mainly due to the adoption of the IoTinternet of things (IoT). To be able to make correct decisions, from those vast amounts of data, the knowledge needs to be extracted. This task is usually done using machine learning algorithms, nowadays very often implemented as neural networks. Fast and well-defined system response time increasingly becomes a more decisive factor in the Quality of Service.

**Figure 1:**
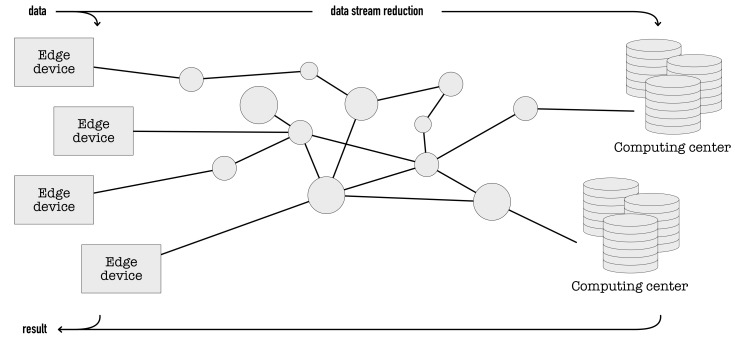
Data flow and processing stages in a standard system setup.

The structure of a conventional data processing and knowledge extraction system is presented in Figure 1, with most of the data processing done in the computing cloud. As a result, the latency of the system response depends on both computing and data-transfer time between edge devices, over the network, and to the data centers. The computing acceleration is possible at every step of the path. The most notable latency decrease can, however, be achieved by relocating as much of the computations into the edge (IoT) devices as possible. Consequently, the amount of data transferred and processed in the upper levels of hierarchy can be significantly reduced.

**Figure 2:**
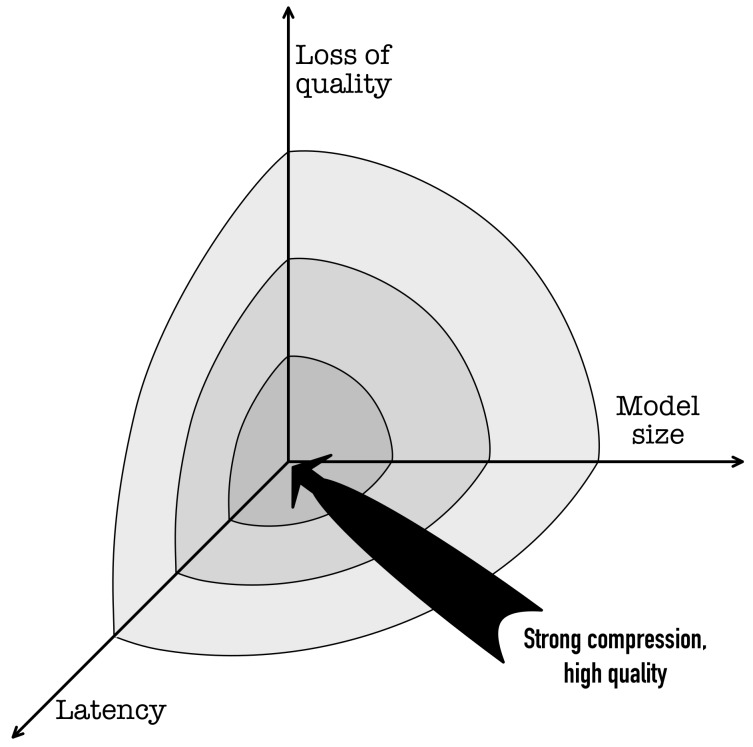
Constrains and demands for Deep Learning models compression.

The maintenance or improvement of the system working parameters, simultaneous with the growing amount of data, and increased expectations regarding the system response time is a complex problem. It was conceptually presented in Figure 2. There are three main dimensions of the compression operation, namely the model size, the latency of the model response, and quality of the processing results delivered by the compressed model. Aggressive compression usually leads to a reduction of model size and processing latency at the expense of the quality of the model performance (e.g., an accuracy drop in the classification set-up) which is undesirable and even not acceptable in many applications. Consequently, the most desirable but unreachable result of the compression operation may be visualized as the coordinate system origin, as presented in Figure 2. Moving towards this location requires deep learning (DL) models to be compressed in a way that does little or no harm to their performance. Furthermore, the compressed modules implemented in the IoT platforms need to be scalable and portable across different devices within the sensor network. This requirement imposes additional conditions on the compression process and makes it even more challenging.

It is worth noting that for over 30 years, Moore’s law determined the progress in computing devices. Thus, the preferred strategy was to wait for a new generation of devices to show up rather than optimizing existing software and platforms. Nowadays, in an era of IoT, multi-core processors, deep learning, and 5G networks, it is better to develop custom solutions which can be employed across various embedded platforms.

Unfortunately, designing, training and modifying DL architectures, created with currently widely available frameworks offering a lot of flexibility and extensive features range, remain in contrast to rigid and well-grounded hardware digital systems design methodology [1,2,3,4]. Bringing those two different worlds together is a challenging endeavor, especially when it comes to creating a methodology and a framework which preserve all the desirable traits of machine learning software.

Popular frameworks provide extensions designed for mobile applications [5,6,7]. However, as they target mobile devices such as smartphones, latency requirements are not very strict. For example, TensorFlow Lite [5] provides performance benchmarks for popular architectures, and the lowest mean inference time is reported for quantized Mobilenet_1.0_224 on iPhone 8 which is 24.4 ms, for other models or on other devices latency is higher.

To accelerate neural models inference many researchers turned to field-programmable gate arrays (FPGAs) [8,9,10]. However, they usually target larger networks, often with image processing in mind. Such architectures are too sizable to be fully unrolled in hardware; therefore, their implementations are based on various types of processing elements with loadable parameters.

Authors of survey [9] put together several neural networks implementations in FPGAs and compared their performance and resource requirements. Usually, the factor that implementations are optimized against is the number of operations per second. Performance is often achieved by processing many samples in a single batch; however, they also report latency for batch size equal to 1, which is desirable for IoT devices working in the real-time regime. Depending on the network model and the device it was run on, the latency varies from a few milliseconds to a couple of hundreds of milliseconds.

LeFlow [8] presents a very interesting usage of Google’s XLA compiler. Authors provide latency results for single layers, and they were able to infer through a small fully connected layer in 380 clock cycles at 267.45 MHz resulting in 1420 ns latency, fully unrolled element-by-element multiplication of two arrays of size 64 took 428 ns.

The most similar work to ours is presented in [10]. The authors use Xilinx Vivado HLS for mapping to FPGA along with their custom supporting code. The tool was benchmarked with a simple four-layer fully-connected model. More details about this experiment can be found in Section 3.4.

The primary pitfall of the commercially available frameworks is the restricted choice of models and the limited ability to modify and extend both the flow and architectures. On the other hand, mapping applications developed by more research-oriented teams suffer from several deficiencies which make them hard for practical application both in terms of the design time and the flow complexity. They usually lack a clear and convenient path of migration between high-level tools used for model development and simulation and low-level software used directly for FPGA mapping. Consequently, all the issues which occur on a platform level which involve model modification, such as running out of available resources and necessity to redesign the model, are very time-consuming and error-prone. Usually, the low-level software was not created with DL models mapping in mind, so there are no routines and mechanisms which facilitate the process.

The main techniques for neural network compression include weights pruning, weights quantization [4,11]. The pruning is done with the aim of the elimination of selected weights by zeroing them, or, in a more sophisticated version, forcing them to acquire specific values so that their histogram is narrow [4]. Employment of pruning before weights quantization allows to significantly improve both the quality of results generated by the compressed model and the efficiency of weights quantization itself. Several pruning methods are described in Appendix A.

The purpose of weight quantization is the conversion from float to fixed-point representation, as required by efficient hardware implementation. Change of the representation from floating-point to fixed-point representation affects the dynamic range of possible representation. However, when the distribution of the weights is unimodal and narrow, it can be adequately represented as fixed-point. It is worth noting that modern FPGAs can handle floating-point operations, but they are very resource-consuming and sub-optimal [12]. Common quantization approaches are described in Appendix B.

Several works [4,13,14] showed that pruning, quantization, and retraining results in much better results in terms of the weights sparsity than just using static operations. The retraining operation allows to shape histogram of weights and dynamically modify them. There is a range of methods which address this task, and it is still a field of intense exploration [15].

The mapping process should account for each of model, data, and the platform profile, and this is a primary goal of the compression procedure. The compression may be done through the reduction of the weights’ bit-width to its limit, while simultaneously retaining the high quality of results produced by the model.

This paper aims to introduce a methodology for mapping neural models to hardware FPGA platforms with a particular focus on IoT devices. The proposed approach may also be used to assess the feasibility of distributed sensors systems concerning resources consumption of individual nodes as well as the latency budget.

We also conducted the experiments with data bucketization (as introduced in [16,17]) which can enable data stream down-scaling between nodes within the system. This down-scaling is done with little or none performance degradation of the model of several modes distributed across devices within the network.

For mapping the model which specified compression parameters to FPGA, we have developed a tool, called DL2HDL (see Supplementary Materials). The tool encapsulates a series of hardware-oriented conversion procedures which enable efficient implementation of neural architecture components (e.g., layers and activation functions).

This paper’s main contributions are as follows:

- A methodology for mapping recurrent neural network-based models to hardware,
- A case studies of the proposed methodology, done in the area of time series analysis (TSA),
- Custom set of scoring metrics, along with multi-objective covariance matrix adaptation evolution strategy (MO-CMA-ES) applying scheme,
- DL2HDL, a publicly available framework to map models written in Python to FPGAs.

The rest of the paper is organized as follows. The Section 2 explains the proposed methodology and describes the mapping tool and FPGA implementation details. In the Section 3 the case study setup and experimental results are described. Finally, the Section 4 contains the discussion and future work directions, while conclusions are presented in the Section 5.

## 2. Materials and Methods

The ultimate goal of the mapping procedure is to stay within the latency budget, even if it incurs some acceptable performance loss. The critical aspect of the process is to mitigate the design timing requirements and resources consumption. Consequently, dedicated strategies and methods are essential.

### 2.1. Architecture of Tensors Processing

There is a set of processing stages in the proposed flow which shape tensors propagated throughout the neural module. In each stage, a different operations’ precision and operands of various bit-width can be used.

**Figure 3:**
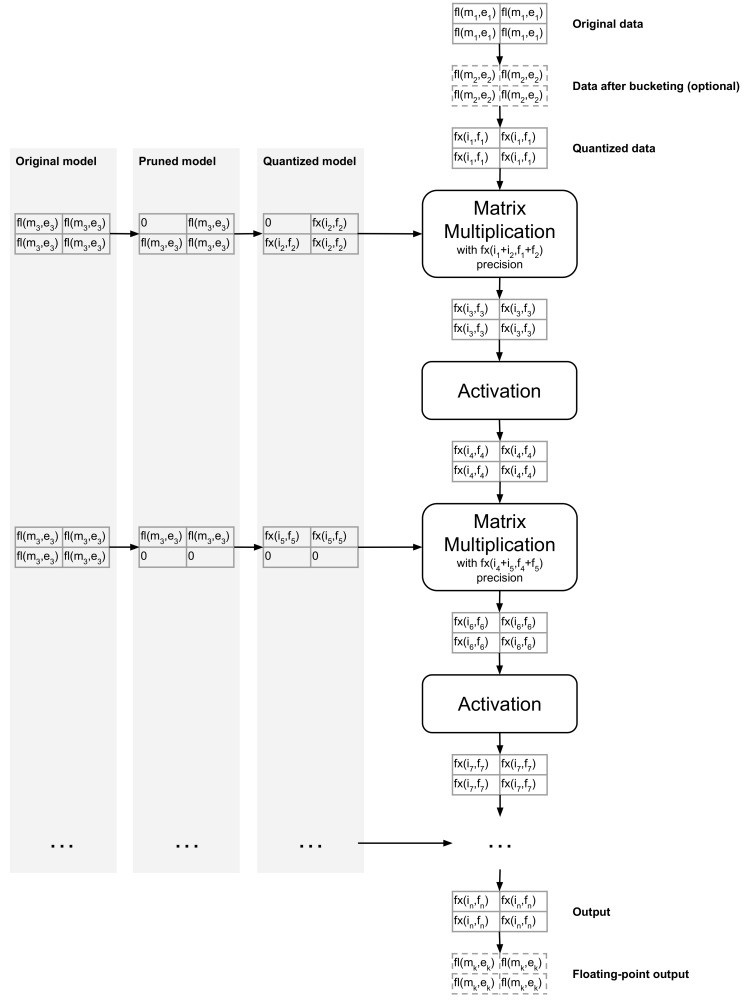
Simplified data flow during inference; fl(m,e) denotes a number in floating-point notation, with *m* bits for mantissa and *e* bits for exponent; fx(i,f) denotes a number in fixed-point notation, with *i* bits for integral part and *f* bits for fractional. In the simplest case $m_{0}=m_{1}=\ldots =m_{k}$, $e_{0}=e_{1}=\ldots =e_{k}$, $i_{0}=i_{1}=\ldots =i_{n}$ and $f_{0}=f_{1}=\ldots =f_{n}$, however those values can be arbitrary chosen to achieve the best resources utilization.

An overview of the proposed processing flow is presented in Figure 3. The high-level sequence of operations is very similar whether the flow in FPGAs after the module was mapped to hardware, or the one performed as an emulation before mapping, done on central processing unit (CPU), is considered.

When entering the module, the original (input) data was subject to optional bucketization and quantized. The bucketization operation has a beneficial impact on the utilization of the available dynamic range of the input data, which was discussed in [16,17]. Then, the data was quantized through mapping to the fixed-point notation. The data quantization mapping schemes were the same as weights quantization ones, presented in Appendix B.

Figure 3 also presents weights pruning and quantization flow. These operations were done in the same way regardless of the platform (CPU or FPGA). In the first step, weights are pruned, and then the quantization operation is performed. If the processing was done on CPU, the quantized weights were stored in the floating-point container. Alternatively, for hardware operations, the quantized weights were directly mapped to FPGA, and the computations were conducted using fixed-point representation.

The core operation in the neural models’ computations was matrix multiplication. When the model compression was emulated on the CPU, the multiplication was done in floating-point precision. On the other hand, in the FPGA flow, the matrix multiplication was done with the precision that prevents overflow, e.g., for two 8 bit arguments fed to single atom multiplication the result occupies 16 bits and was truncated to eight bits (upper eight bits were taken). Consequently, to precisely emulate the hardware flow on CPU, after each matrix multiplication operation, the result should be quantized. This operation is called the activations quantization. It is worth emphasizing that all operations within the hardware platform (FPGA) were done using fixed-point, and the activations quantization can be perceived as being done automatically.

During the emulation, the activations quantization effect can be observed through the performance degradation (e.g., accuracy drop in the classification task). When the performance degradation is too severe, it means that the activations quantization was too aggressive, i.e., too few bits were allocated for the propagation of the activation between the processing stages of the module. If activations quantization was applied during emulation, the module’s output can be extracted from FPGA and mapped back to the floating-point, and then directly compared with the CPU results.

In the proposed mapping scheme, we did not incorporate activation quantization in the flow but used functional simulation of the hardware description language (HDL) code. This omission is possible since our flow is equipped with the simulation step. By examination of the simulation results, we can determine if the overflow occurs. When it happens, the bit-width allocated for the activations should be increased.

### 2.2. Mapping Strategies

Different paths may be taken to map a deep learning model to platforms with limited resources. The process is complicated and depends on preliminary constraints of a particular design. Two different strategies may be adopted when it comes to the starting point of the mapping flow, the choice of which depends whether we start from scratch or with a pre-trained model. The first strategy leaves much more freedom for the mapping procedures to be applied since the architecture of the model is not fixed at this point and can be modified. On the other hand, when it comes to a mapping of a pre-trained model, we are much more limited concerning the number of available tools that can be used in the process, since the architecture and the internal structure should remain as intact as possible. In practice, it is hardly ever achievable, and modifications such as quantization, pruning, or compression are essential to reduce response latency and memory footprint of the model.

We propose a series of strategies based on our experience in hardware design, deep learning architectures, and FPGA optimization. Since FPGAs may be considered as highly flexible among application-specific integrated circuits (ASICs), general-purpose graphics processing units (GPGPUs), CPUs, and other dedicated platforms [18,19], the presented strategies may be tailored for other hardware as well.

**Figure 4:**
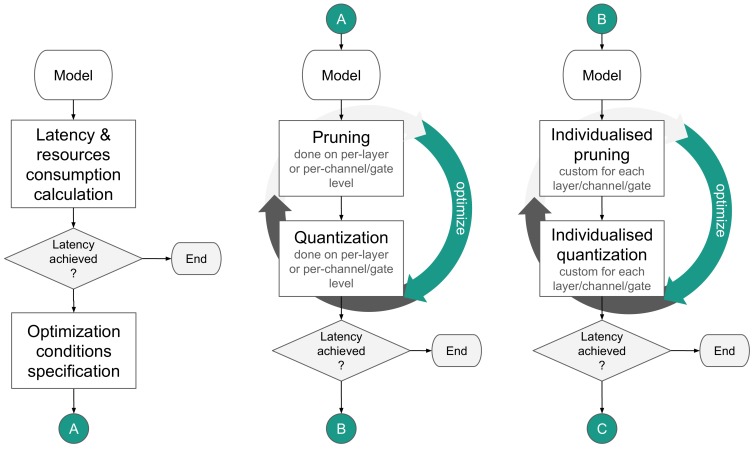
Strategies for mapping a pre-trained model.

**Figure 5:**
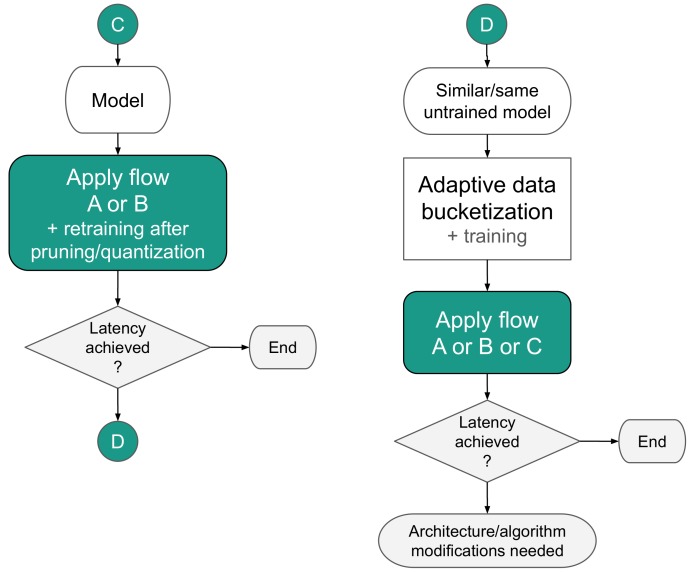
Advanced strategies for model mapping.

The strategies are split into two main paths. The first one is addressing the flow of a pre-trained model (Figure 4). The second one covers a scenario where a designer starts from the beginning with all the freedom to create a model, which however has to be optimized with hardware constraints in mind (Figure 5).

Before any strategies are attempted, the pre-trained model should be examined concerning incurred latency and resources consumption. The initial calculations should address the border scenario, i.e., the least amount of memory the *L*-layered model may occupy, assuming the weights are compressed to one bit and 95% of coefficients are removed (Equation (1)).

(1) $$\mathrm{memory}=\sum_{i=0}^{L-1}\mathrm{size}({\mathrm{layer}}_{i})*1*0.05.$$

Such quantization and pruning settings are, of course, very optimistic and hardly ever achievable. Consequently, if border conditions analysis shows that the model still exceeds available resources, its architecture should be changed or a bigger FPGA chip should be used.

In the next step, the baseline results should be obtained. It can be done by a simple mapping operation performed after changing the data format to fixed-point. The format change was needed since even though the float-point representation absorbs the same amount of memory (e.g., 32 bit), it leads to much more expensive arithmetic operations. Usually, it turns out after the synthesis and implementation for FPGAs that the model exceeds available resources of the chip. After the compression potential of the model was analyzed, a suitable set of strategies and parameters for the next stages can be chosen.

Ehile two basic proposed flows, A and B, are very similar, the second one is, however, more computationally demanding, therefore it is advisable to start with scenario A if possible. When applying both pruning and quantization, which was the most common case, the pruning should be applied first. It is because quantization granulates the weights search space of the optimal point, making the pruning process harder to converge. The pruning and quantization operations are usually applied on the per-tensor level. However, for some models, especially the ones equipped with depth-wise/point-wise layers (e.g., Mobilenet, Resnext, Xception) performing pruning and quantization operations on per-tensor level can lead to significant degradation of performance [20]. Therefore, the higher granularity of the operations (channel or gate level) is essential for better convergence of pruning and quantization operations.

As incurred latency is affected both by the number of required operations and their precision, the first stage of model mapping procedure should allow to quickly obtain a set of pruning and quantization parameters’ combinations for further (and more time-consuming) tests. To this effect, we propose to apply a software pruning/quantization simulation and some form of multi-objective optimization. For large datasets, the optimization can be performed using a small, but representative test dataset (e.g., 5% to 10% of original), and final results should be validated on the full test dataset. The optimization objectives should include keeping the model quality as close to original as possible, ensuring the biggest overall model weights’ sparsity, and bringing the precision down as much as possible. The analysis of the original model resources consumption can help to define the range of the optimization parameters, e.g., it may turn out that to fit the model into the required hardware reaching a specific sparsity or quantization level is required.

The generated parameters’ combinations suitability for further tests may need to be further examined, e.g., to remove all combinations that yield the model with quality drop above the acceptance threshold, usually ≈1 percentage point (p.p.). It may also be beneficial to run the inference with the full testing dataset and verify the model quality. Once the candidates were selected, the pruned and quantized models were once again mapped to FPGA for the examination of incurred latency. If the latency is satisfactory, the procedure is over; else flow B can be used. It may also turn out that the flow B better addresses the distribution of the compressed model across the IoT platforms comprised of multiple nodes with limited and different resources.

In the flow A, the pruning and quantization parameters were chosen for the whole model (e.g., the precision of N bits) and then applied per-tensor (e.g., prune X% of each tensor, find the number of integral/fractional bits that will best represent this tensor). In scenario B, the pruning and quantization parameters are chosen and applied on the per-tensor basis, i.e., each tensor can have different precision and pruning conditions than the other. This individualization can potentially yield even better results, but is much more computationally intensive, since the number of optimized parameters grows with the number of layers.

The next two mapping methods cover scenarios when the model is created from scratch or can be retrained, and the flexibility of modification is left to the designer. In scenario C, the model is interchangeably quantized (using flows A or B) and retrained to regain performance lost in the process and shape the coefficients. Especially trained quantization [21,22] allows shaping coefficients in a way which make a whole model more pruning-prone. If the expected latency is not satisfied, procedure D can be tried.

Procedure D differs from the previously presented scenarios since it incorporates input data bucketization. As such, it is suitable mostly for classification tasks. In the first step, the data should be bucketized, and the model trained. We recommend the adaptive bucketization procedure, which takes the best advantage of the dynamic range of data representation (please See Section 2.1 and Appendix A of [17] and Section 4.2 of [16] for details). This process is arbitrary and should be monitored concerning the performance of the model.

It is worth mentioning that using data bucketization makes the data more susceptible to compression, which in turn may result in the data throughput increase within the system. Furthermore, during experiments, we have noticed that data bucketization may also positively impact model compression (see Section 3.3.5). This effect is caused by the distribution of the input data being affected by a profile of the model weights.

As a second stage of the procedure D, scenario A or B was followed. It those steps fail to meet the latency budget again, scenario C was applied. As the final step, when all those procedures failed to satisfy latency constraints, model modification was recommended. This case was typical when it comes to DL models binarization [11,23] because the network structure was deprived of most information carriers. In such a scenario, after all the operations were applied, the model was extended with few more layers and retrained. The procedure was applied until the performance close the original was reached.

### 2.3. Analyzed Model—LSTM

The proposed methodology can be applied to various models; however, in our work, we focused on models that can be used for predictive maintenance or anomaly detection, in particular, the long short-term memory (LSTM) networks.

All the tested models utilized LSTM cells and dense units. Each of the LSTM layers comprised of three tensors (*W*—input connections weights, *U*—recurrent connections weights and *b*—bias). Each of the LSTM layer tensors can be further deconstructed into four tensors: *i* (input), *f* (forget), *c* (cell state) and *o* (output) ones. For input tensor *x* at time *t* the LSTM layer output $h_{t}$ can be calculated as in Equations (2)–(6), with $c_{0}=0$, $h_{0}=0$, σ being the hard sigmoid activation function and the ∘ operator denoting the element-wise product.

(2) $$i_{t}=\sigma \left(W_{i}x_{t}+U_{i}h_{t-1}+b_{i}\right)$$

(3) $$f_{t}=\sigma \left(W_{f}x_{t}+U_{f}h_{t-1}+b_{f}\right)$$

(4) $$c_{t}=f_{t}\circ c_{t-1}+i_{t}\circ \tanh\left(W_{c}x_{t}+U_{c}h_{t-1}+b_{c}\right)$$

(5) $$o_{t}=\sigma \left(W_{o}x_{t}+U_{o}h_{t-1}+b_{o}\right)$$

(6) $$h_{t}=o_{t}\circ \tanh\left(c_{t}\right)$$

A dense layer can be described by two tensors; *W*—input connections weights and *b*—bias. The parameter ϕ denotes the activation function—we have used softmax for classification tasks and linear activation for regression. A dense layer output can be calculated as follows:

(7) $$o_{t}=\phi \left(Wx_{t}+b\right).$$

For calculation of the total number of model parameters, the size of each tensor needs to be found. If *k* is the number of input features, *m*—output features, and *n*—LSTM cells, the tensor sizes can be calculated as in Equation (8):

(8) $$\begin{array}{cc} \mathrm{size}(W_{LSTM}) & =\mathrm{size}\left(W_{i}\right)+\mathrm{size}\left(W_{f}\right)+\mathrm{size}\left(W_{c}\right)+\mathrm{size}\left(W_{o}\right)\\ & =k\;\cdot \;n+k\;\cdot \;n+k\;\cdot \;n+k\;\cdot \;n=4\;\cdot \;k\;\cdot \;n,\\ \mathrm{size}(U_{LSTM}) & =\mathrm{size}\left(U_{i}\right)+\mathrm{size}\left(U_{f}\right)+\mathrm{size}\left(U_{c}\right)+\mathrm{size}\left(U_{o}\right)\\ & =n\;\cdot \;n+n\;\cdot \;n+n\;\cdot \;n+n\;\cdot \;n=4\;\cdot \;n^{2},\\ \mathrm{size}(b_{LSTM}) & =\mathrm{size}\left(b_{i}\right)+\mathrm{size}\left(b_{f}\right)+\mathrm{size}\left(b_{c}\right)+\mathrm{size}\left(b_{o}\right)\\ & =n+n+n+n=4\;\cdot \;n,\\ \mathrm{size}(W_{Dense}) & =n\;\cdot \;m,\\ \mathrm{size}(b_{Dense}) & =m. \end{array}$$

It is worth noting that in the case of multi-layer networks, the number of previous layer’s LSTM cells becomes the number of input features for the next layer. For example, a two-layer LSTM network, with n=[8,4] with k=3 input features (data channels) and m=2 output features (classes; e.g., anomaly/non-anomaly) would yield following values:

(9) $$\begin{array}{cc} \mathrm{size}(W_{LSTM_{1}}) & =4\;\cdot \;k\;\cdot \;n_{1}=96,\\ \mathrm{size}(U_{LSTM_{1}}) & =4\;\cdot \;n_{1}^{2}=256,\\ \mathrm{size}(b_{LSTM_{1}}) & =4\;\cdot \;n_{1}=32,\\ \mathrm{size}(W_{LSTM_{2}}) & =4\;\cdot \;n_{1}\;\cdot \;n_{2}=128,\\ \mathrm{size}(U_{LSTM_{2}}) & =4\;\cdot \;n_{2}^{2}=64,\\ \mathrm{size}(b_{LSTM_{2}}) & =4\;\cdot \;n_{2}=16,\\ \mathrm{size}(W_{Dense}) & =n_{2}\;\cdot \;m=8,\\ \mathrm{size}(b_{Dense}) & =m=2, \end{array}$$

summing up to 384 parameters in first LSTM layer, 208 in the second and 10 in the dense layer, with the total number of model parameters equal to 602.

Applying the flow A of the presented methodology to the LSTM network means that the pruning and quantization parameters are selected globally for the model, and then applied for each of the *W*, *U*, and *b* tensors. For example, when ‘hist_amount’ pruning method was used and pruning fraction =0.1, it is ≈10% of each tensor that was pruned, and not some small fraction in one layer and much higher in another.

Flow B, on the other hand, was designed to realize something akin to that second scenario. Its objective was, in our case, finding the best pruning and quantization parameters for each of the *W*, *U*, and *b* tensors individually, potentially resulting in lower resources consumption (e.g., when some of the tensors can be more aggressively compressed than in flow A) or better quality (e.g., retaining the high precision representation where it is needed and dropping it elsewhere). For the two-layer network mentioned above, this means that instead of finding the single pruning fraction value, it needs to optimize eight of them; similarly for the quantization bits.

### 2.4. Optimization Strategy—MO-CMA-ES

In this work, we deal with a range of relatively small number of parameters, but quite diverse. There were also several criteria (e.g., the sparsity of the model, model performance) which were simultaneously taken into account during the optimization process. This multiplicity makes it very challenging to define a single descriptive objective function. Lack of a well defined objective function or an inability to provide such may be prohibitive for using simple optimization methods or heuristics. Furthermore, the parameter space is non-convex and not differentiable; thus, the gradient-based optimization methods may not be employed. Utilization of naive grid or random search approaches does no lead to satisfactory results because those procedures do not account for a complex nature of the parameters space and do not improve in the process. Consequently, a very long examination time or a very dense grid of a parameters space would be required, which is prohibitive, especially for large models where the computing time of each instance is long.

This leaves us with black-box optimization techniques. In this work, we have decided to utilize evolutionary approach. There are many of such techniques [24], but all of them may be formulated as an algorithm that delivers a set of candidate solutions to evaluate the optimization task (see Algorithm 1).

**Algorithm 1** Basic evolutionary strategy. **while**
*true*
**do**   *token*, *solution* ← *sampler*.ask()▹ ask for candidate solution   *loss* ← evaluate(*solution*)▹ evaluate the given solution   *sampler*.update(*token*, *loss*)▹ give fitness result back to ES   *sampler*.update(*token*, *loss*)   **if**
*loss* < REQUIRED_LOSS **then**     **break**   **end if** **end while**

The straightforward approach of evolution strategy (ES) is based on using a standard distribution with initially defined mean and variance. In each step, the very best value from the previous step of the procedure and use it as a new mean. This approach has severe limitations and is prone to get stuck in a local minimum. There is also a whole range of genetic algorithms (GAs) which introduce mechanisms such as crossover recombination and mutations. GAs help to maintain a diversity of a set of candidate solutions and perform better than the simpler algorithms. A whole range of improved GAs was also developed, such as Enforced SubPopulations (ESP) [25], and NeuroEvolution of Augmenting Topologies (NEAT) [26], which use clustering of similar solutions to maintain better diversity of the population.

A limitation of simple ESs and GAs is taking a fixed value of standard deviation noise parameter. This premise affects the efficiency of the explore-and-exploit mechanism operating in the solution space. When an algorithm is close to the desired solution with high confidence, it should exploit it; on the other hand, in the exploration phase, the algorithm should penetrate as ample candidate solution space as possible.

The covariance matrix adaptation evolution strategy (CMA-ES) is a modern single objective ES, introduced for the first time in [27], and later improved in [28,29]. It turned out that it works very well in a wide range of problems in a continuous domain.

**Figure 6:**
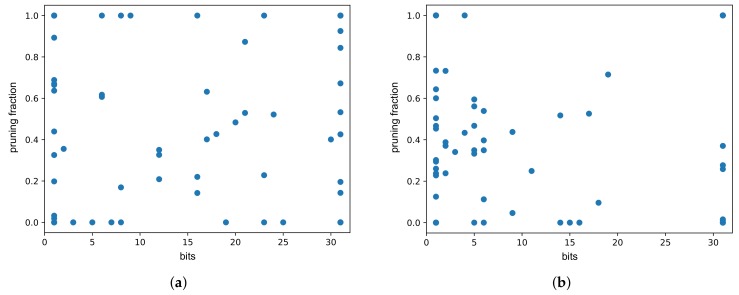
Sample multi-objective covariance matrix adaptation evolution strategy phases. (**a**) Explore—algorithm samples the whole search space; (**b**) exploit—algorithm starts to converge to the best solutions.

The CMA-ES uses a number of best solutions from the current generation to calculate both mean and standard deviation of the parameters being optimized. The change of the standard deviation value allows to adjust the size of the search space, i.e., whether algorithm focuses on exploitation or exploration, as can be seen in Figure 6. Those re-calculated values are then used to generate the candidate solutions for the next generation, with parameters sampled from a multivariate normal distribution.

One of the beneficial properties of CMA-ES is the rate at which good approximations of the global minimum can be found. It is also resistant to using the incorrect initial set of parameters due to its self-adaptive nature.

There is an extension of CMA-ES for multi-objective optimization denoted as MO-CMA-ES [30]. Since MO-CMA-ES operates on multiple objectives, it yields Pareto fronts as a result (Algorithm 2). The Pareto front is a set of results which cannot be improved any further i.e., improvement of any of the objectives can be made only at the expense of the others. The Pareto fronts are also used in the process of the next candidate solution selection.

A detailed description of CMA-ES and MO-CMA-ES is beyond the scope of this paper, however we encourage the reader to look into [24,27,28,29,30] for more information.

**Algorithm 2** Multi-objective evolutionary strategy. **for**
*step* ← to *max_steps*
**do**   *token*, *solution* ← *sampler*.ask()▹ ask for candidate solution   *loss* ← evaluate(*solution*)▹ evaluate the given solution   *sampler*.update(*token*, *loss*)▹ give fitness result back to ES **end for** *results* ← *sampler*.results() *first_front* ← get_first_pareto_front(*results*)

To optimize pruning and weights quantization parameters we used MO-CMA-ES provided by the chocolate package [31]. We optimized four basic parameters: pruning method, pruning fraction, quantization method, and quantization bit-width. The number of parameters increased to 2+2∗L with the number of layers *L* when the individualized approach was used. Evaluated pruning methods are described in Appendix A, and quantization methods are explained in Appendix B.

We used three optimization criteria (designed for minimalization):

1. Quality score, where ${\mathrm{qual}}_{\mathrm{orig}}$ is the original model’s quality measure, preferably in [0,1] range, ${\mathrm{qual}}_{\mathrm{pq}}$ is the quality after weights pruning and quantization, and allowed_drop=0.01 is the allowed quality drop. If qualityscore<1, the pruned and quantized model’s quality is considered to be satisfactory.
  (10) $$\mathrm{quality}\;\mathrm{score}=\frac{{\mathrm{qual}}_{\mathrm{orig}}-{\mathrm{qual}}_{\mathrm{pq}}}{\mathrm{allowed}\;\mathrm{drop}}.$$
  When using a quality measure that is designed to be minimized (such as root-mean-square error (RMSE)), the quality score is additionally negated.
2. Sparsity score, where *N* is the number of model weights, and $w_{i}$ is the *i*-th weight value.
  (11) $$\mathrm{sparsity}\;\mathrm{score}=1-\frac{1}{N}\sum_{i=0}^{N-1}\left(w_{i}==0\right)$$
3. Used bits score, where *N* is the number of model weights, and bit-width $\left(w_{i}\right)$ is the *i*-th weight bit-width after quantization. Used bits score is adjusted to fall into [0,1) range, assuming the maximum allowed bit-width after quantization is 31 bit.
  (12) $$\mathrm{used}\;\mathrm{bits}\;\mathrm{score}=\frac{1}{N\;\cdot \;32\;\mathrm{bit}}\sum_{i=0}^{N-1}\mathrm{bit-width}\;\left(w_{i}\right).$$

As presented in Algorithm 2, the optimization was run for a fixed number of *max steps*, calculated according to the Equation (13). The number of parents used to generate the candidates was mu=30.

(13) maxsteps=numberofparameters∗numberofoptimizationcriterions∗mu∗10.

### 2.5. Mapping Tool

As mentioned in Introduction, there are many challenges associated with hybrid model mapping, such as inter-tool format translation, arbitrary precision emulation, and incorporation of pruning and quantization on all the processing levels. Rather than enumerating and presenting all of them, it is better to provide a set of requirements which a decent and useful framework should meet. They are based on our experience of many years of using high-level languages (HLLs) for hardware design such as OpenCL, MitrionC, ImpulseC, CataputC as well as DL models training. Primarily educative in terms of experience was our work [32], which led us to an idea and an architecture presented in this paper.

A high-level tool for mapping Machine Learning models to hardware, especially FPGAs should:

- provide the ability to migrate, map and test using a single set of tests at all levels of the project description abstraction (from the meta description on the top layer, through the intermediate format up to HDL). This ability is especially important if there are changes in the notation of the data structure at particular levels of the description,
- allow parametrizing modules on a high level of description,
- provide a mechanism for arbitrary data representation and modification along with all the associated arithmetic operations,
- enable of simulation at all the development levels, preferably using the same tests, or provide mechanisms which generate test-benches for hardware simulations,
- come with a library of predefined DL modules comprising a set of basic application programming interfaces (APIs) for developing new custom elements,
- include HDL generation back-end which preserves the original architecture of the model, so the debugging and analysis on the hardware level is facilitated.

**Figure 7:**
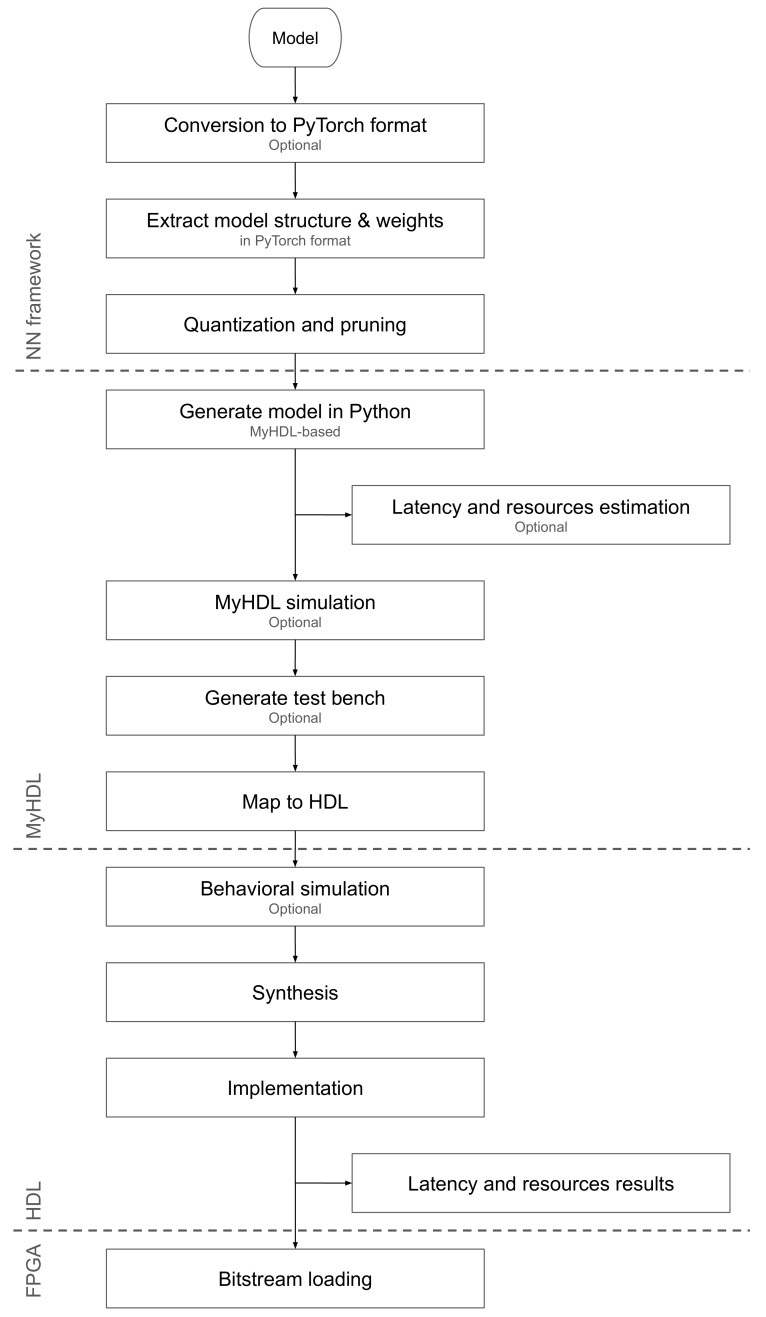
DL2HDL flow.

We treated the directions presented above as guidelines in the process of designing our DL2HDL (see Supplementary Materials). The block diagram of the tool flow is presented in Figure 7.

In the first step of the process, a deep learning model was trained in Keras or PyTorch. Since the main flow is in PyTorch, a model trained or provided in Keras is converted to PyTorch. The conversion tool was embedded within the flow. The PyTorch was chosen because it is a dynamic-graph-based framework, which made it much easier for debugging and instrumenting the code. It was especially vital during the initial stages of development of the flow.

Once the DL model was trained, quantized and pruned, the three actions were taken:

- appropriate components from the custom DL library are picked and embedded in the model,
- MyHDL wrapper around the DL learning components was generated,
- MyHDL test files were generated to be used for verification of the code this stage.

The custom library of the DL components was created along with a tool which populates a DL model with the elements from the library. Currently, the library contains the following components:

- a set of activation functions: *relu*, *sigmoid*, *tanh* and *softmax*
- a set of layers: *linear*, *conv1D*, *conv2D*,
- LSTM,
- *maxpool*.

All the layers were parameterizable, which enabled smooth integration to form a complete model. In addition to the layers, sample complete models based on recurrent neural network (RNN) and convolutional neural network (CNN) were available.

Next, once the model was compiled HDL was generated using the to_HDL conversion tool based on MyHDL package [33]. From this point in the flow, a standard FPGA flow was followed, which is composed of functional simulation, synthesis, and implementation. As the final step, a bitstream was generated and uploaded to FPGA.

It is worth noting that at any stage of this flow, it was possible to revert and redo the step before. This option was per the principles of proposed methodology because it allows for redoing the operations many times before the satisfactory latency is met.

### 2.6. FPGA System Architecture

**Figure 8:**
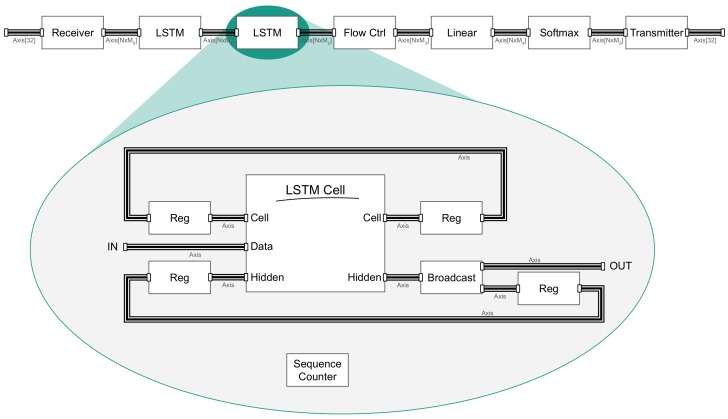
Field-programmable gate array (FPGA) network implementation schematic. *N*—bit width, *M*—# of elements in a feature map.

Figure 8 presents the schematic of the network implemented using DL2HDL tool. All component are connected using AMBA AXI4-Stream interface [34], which facilitates easy modifications. Data transfer to and from the network is managed by receiver, and *transmitter* blocks. In our implementation, a receiver collected serial data using 32-bit wide interface and when full feature map is received, transmits it as one data word. For example, if input data for the LSTM is a set of values from four analog-to-digital converters (ADCs) then the output word width was 4× ADC data width.

This approach allows for full unroll of operations in subsequent blocks. The transmitter works similarly but in the opposite direction. However, thanks to the modular approach and standard interface, receiver and transmitter blocks can be easily replaced to match the desired interface. LSTM layers work on a series of data, but the linear layer needs a single sample, and for that purpose, the flow control block was introduced. It counted the sequence samples and passed only the last one to linear layer; other connection schemes can also be implemented. Inside the LSTM, apart from an LSTM cell, additional registers were placed to ensure synchronization of passing data in temporal dimension, additional sequence counter control clearing and setting these registers to zero input at the beginning of each new sequence.

The LSTM cell was built according to the Equations (2)–(6). First, the linear operations were performed, and gates were concatenated for input and hidden state so that only two linear modules were required. Then the data was split for separate activations, and all element-wise operations were performed. Single data word contains a complete feature map; therefore, element-wise operations can be fully unrolled. As they execute relatively simple operations, additional registers were not necessary; as a result, element-wise operations do not introduce latency in terms of clock cycles, only indirectly in logic complexity between modules. However, during development, the tests showed that the linear modules have a large impact on final latency. Hyperbolic tangent was implemented as a linear approximation, which is further discussed in Appendix D, and hard sigmoid was implemented similarly.

**Figure 9:**
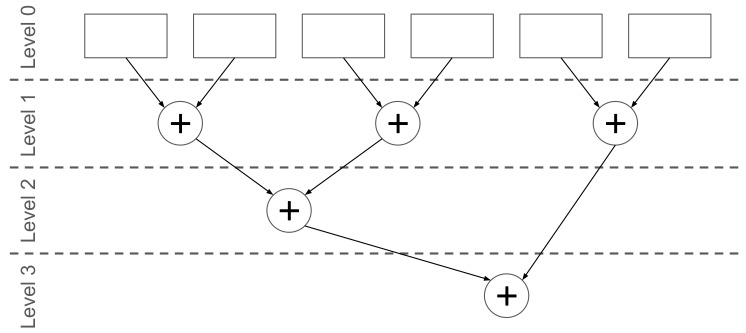
Summation scheme in linear layer.

In linear module operations are split for each output unit, then multiplication is fully unrolled. The results are accumulated using tree addition presented in Figure 9. Summation operation proved to be time-consuming in terms of logic complexity, and it is necessary to insert registers between summation levels for bigger layers. For modern Xilinx devices, it is enough to insert registers every two to three levels to bring logic complexity close to the rest of the design; this parameter can be easily changed. Multiplication results were in fixed point format; summation result was also a fixed point, which introduces the risk of overflow for narrow representations. This effect was mitigated by the distribution of results concentrated on both the positive and negative side of zero; however, to ensure proper operation data width need to be verified during a simulation on training data set. Components inside the LSTM cell were also using AXI4-Stream interfaces to communicate.

Summation in softmax function was implemented similarly as in the linear layer. Additionally, there is a choice between two exponent implementations: calculated approximation, and a look-up table. Look-up table implementation was significantly faster, but its size grows with data width, therefore for wider data approximation scheme should be selected. In most of our experiments, the look-up table proved to give better results.

We have noticed that using the same bit-width for data, weights, and activations representation is, in most cases, wholly sufficient and optimal. Using smaller bit-with may lead to overflow. On the other hand, using a larger bus size is sub-optimal in terms of resources consumption of an FPGA.

## 3. Results

To present and validate the methodology, we have examined three datasets using ’analysta’, our time series analysis framework (see Supplementary Materials). The first one is the International Airline Passengers data from the Time Series Data Library (TSDL) [35]. It was used in a toy example, depicting selected aspects of the methodology. The [IAP] tag was used in figures’ and tables’ captions for easier identification of the related results.

The second dataset contains PM_2.5_ pollution data of the US Embassy in Beijing in conjunction with meteorological data from Beijing Capital International Airport [36]. The model objective in this example was to predict the pollution value. The related results are marked with the [PM2.5] tag.

The third, most extensive use case was based on the experiments conducted in [17], which was focused on exploring the influence of history length and various data quantization schemes on the models’ performance. The data we used came from the post mortem (PM) database of European Organization for Nuclear Research (CERN) Large Hadron Collider (LHC), representing the state of the superconducting magnets. The figures and tables containing related results are marked with the [LHC] tag.

It may be beneficial to estimate and compare the resulting model size with original, uncompressed one to interpret the compression results. For model with *N* weights $w_{i}\in W$, its approximate size after pruning and linear quantization can be calculated as follows:

(14) $$\mathrm{compressed}\;\mathrm{model}\;\mathrm{size}=\sum_{i=0}^{N-1}\mathrm{bit-width}\;\left(w_{i}\right)\;\mathrm{if}\;w_{i}<>0.$$

This value can then be compared with original size, calculated as N·32 bit:

(15) $$\mathrm{percentage}\;\mathrm{of}\;\mathrm{original}\;\mathrm{model}\;\mathrm{size}=\frac{100\%*\mathrm{compressed}\;\mathrm{model}\;\mathrm{size}}{N\;\cdot \;32\;\mathrm{bit}}.$$

### 3.1. International Airline Passengers

To illustrate the selected aspects of the methodology, we used the International Airline Passengers dataset [35]. It contains the number of international airline passengers in thousands per month, collected between January 1949 and December 1960, and has 144 data points in total, first 92 of which we used for training, 23 for validation and the remaining 29 for testing. The history window length was set to 5, and batch size was =1.

#### 3.1.1. Initial Conditions Analysis

The network we used contained n=4 LSTM cells and a single dense unit, with a single input (k=1) and a single output feature (m=1). Total number of parameters, with tensor sizes calculated according to Equation (8), was 101. The uncompressed model memory usage, assuming 32 bit representation, was therefore 404 B. The hypothetical memory usage after extreme compression can be calculated according to Equation (1):

(16) memory=101∗1bit∗0.05≈5bit≈1B.

#### 3.1.2. Pruning/Quantization Optimization

The optimization was done according to flow A—finding per-model weights pruning and quantization parameters. Due to the small size of the original dataset, the optimization process could use it as a whole. The RMSE (see Equation (A14) in Appendix C) was used as a quality measure, resulting in a following quality score:

(17) $$\mathrm{quality}\;\mathrm{score}=\frac{{\mathrm{RMSE}}_{\mathrm{pq}}-{\mathrm{RMSE}}_{\mathrm{orig}}}{0.01}.$$

**Figure 10:**
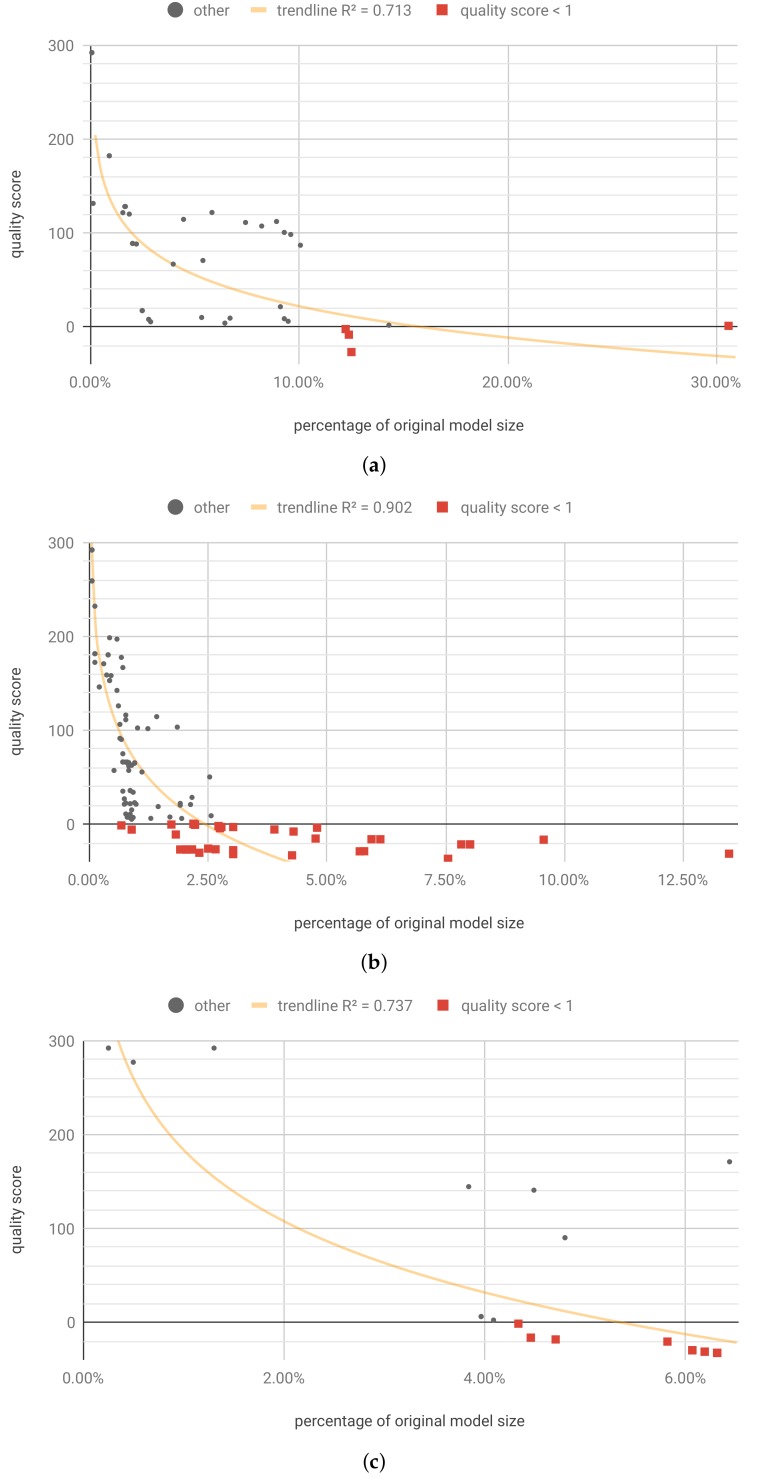
[IAP] Unique first Pareto front results. (**a**) Flow A: per-model pruning and quantization. trendline(x)=−89.1−48.2lnx; (**b**) Flow B: individualized pruning and quantization. trendline(x)=−275−74lnx; (**c**) Flow C-A: pruning and quantization with single retraining epoch. trendline(x)=−320−109lnx.

Optimization was run for 3600 steps and resulted in 49 unique Pareto optimal solutions, four of which retained the required qualityscore<1. The results are shown in Table 1 and Figure 10a. Figure 10a shows that the best result results range 12.22% to 12.50% with size of 4 to 5 bits. It is worth noting that the biggest contribution came from quantization, and the sparsity level due to pruning was relatively low. This sparsity level resulted from a small size of the model, which cannot be pruned as effectively as large models.

#### 3.1.3. Individualized Pruning and Quantization

The individualized weights pruning and quantization optimization (flow B) was run for 10,800 steps. Its aim was to find optimal pruning fraction and quantization bits for each of the *W*, *U* and *b* tensors. It resulted in 192 unique Pareto solutions, 52 of which retained the required quality. While the pruning fraction in each of those solutions was different, only 37 different values of *quality score* and *sparsity score* were yielded, with small values differences having no impact on the final result. Those 37 representative results are shown in the Table A2 (Appendix E) and Figure 10b.

It is worth noting that in this case, the individualized pruning and quantization led to much better results than the per model approach (flow A). This is reflected in Table A2, where the lowest compressed model size is 0.68% of the original. It is worth noting that for the best case in Table A2 sparsity is 78.85% and 1 to 3 bits are used for most of weights representation.

#### 3.1.4. Pruning and Quantization with Retraining

For optimization with retraining, we have limited the range of the optimized parameters to the number of bits ∈[4,31] and pruning fraction ∈[0,0.5). We have applied the retraining procedure using keras.optimizers.SGD (lr = 0.0001, momentum = 0.0, decay = 0.0, nesterov = False) and weights masking. The masking means that the model weights that were pruned were discarded from the training process in the next iteration. Optimization was run for 3600 steps and resulted in 44 unique Pareto optimal solutions, 30 of which retained the required qualityscore<1. In all of those 30 solutions, the ‘thq’ was selected as the quantization method, with the number of bits =4, and ‘simple’ pruning method was chosen. Similarly to the individualized optimization, the pruning fraction in each of those solutions was different, however only seven different values of *quality score* and *sparsity score* were yielded. Those seven representative results are shown in Table A3 (Appendix E) and Figure 10c. It can be seen that for this example, the retraining positively impacts the model compression limits.

### 3.2. Beijing PM_2.5_ Pollution

This dataset contains hourly readings of the PM_2.5_ concentration from the US Embassy in Beijing, combined with the meteorological data from Beijing Capital International Airport [36], and includes missing data. Each sample contains 13 attributes, including date of the reading, hour, PM_2.5_ concentration ($\mu g/m^{3}$), dew point (°C), temperature (°C), pressure (hPa), combined wind direction, cumulated wind speed ($ms^{-1}$), cumulated hours of snow and cumulated hours of rain.

The combined wind direction is expressed as one of five broad categories:

1. northwest (NW), which includes W, WNW, NW, NNW, and N;
2. northeast (NE), which includes NNE, NE, and ENE;
3. southeast (SE), which includes E, ESE, SE, SSE, and S;
4. southwest (SW), which includes SSW, SW, and WSW;
5. calm and variable (CV).

The gaps in the data shorter than 12 h were interpolated. Longer gaps split the data into 37 variable-length series, containing 42,168 samples in total. Series were then assigned to training, validation and testing sets, maintaining roughly a 64-16-20 split. The model task was to predict the value of the PM_2.5_ concentration. For experiments, the history window length was set to 72, with batch size =1024.

#### 3.2.1. Initial Conditions Analysis

The network we used contained 16 LSTM cells and a single dense unit, for a total of 1873 parameters. The hypothetical memory usage after extreme compression is:

(18) memory=1873∗1bit∗0.05≈94bit≈12B.

#### 3.2.2. Pruning/Quantization Optimization

Optimization was run for 3600 steps. Since the dataset is quite small, it was used whole during the optimization process. Similarly to the International Airline Passengers example, the RMSE was used in a *quality score*.

**Figure 11:**
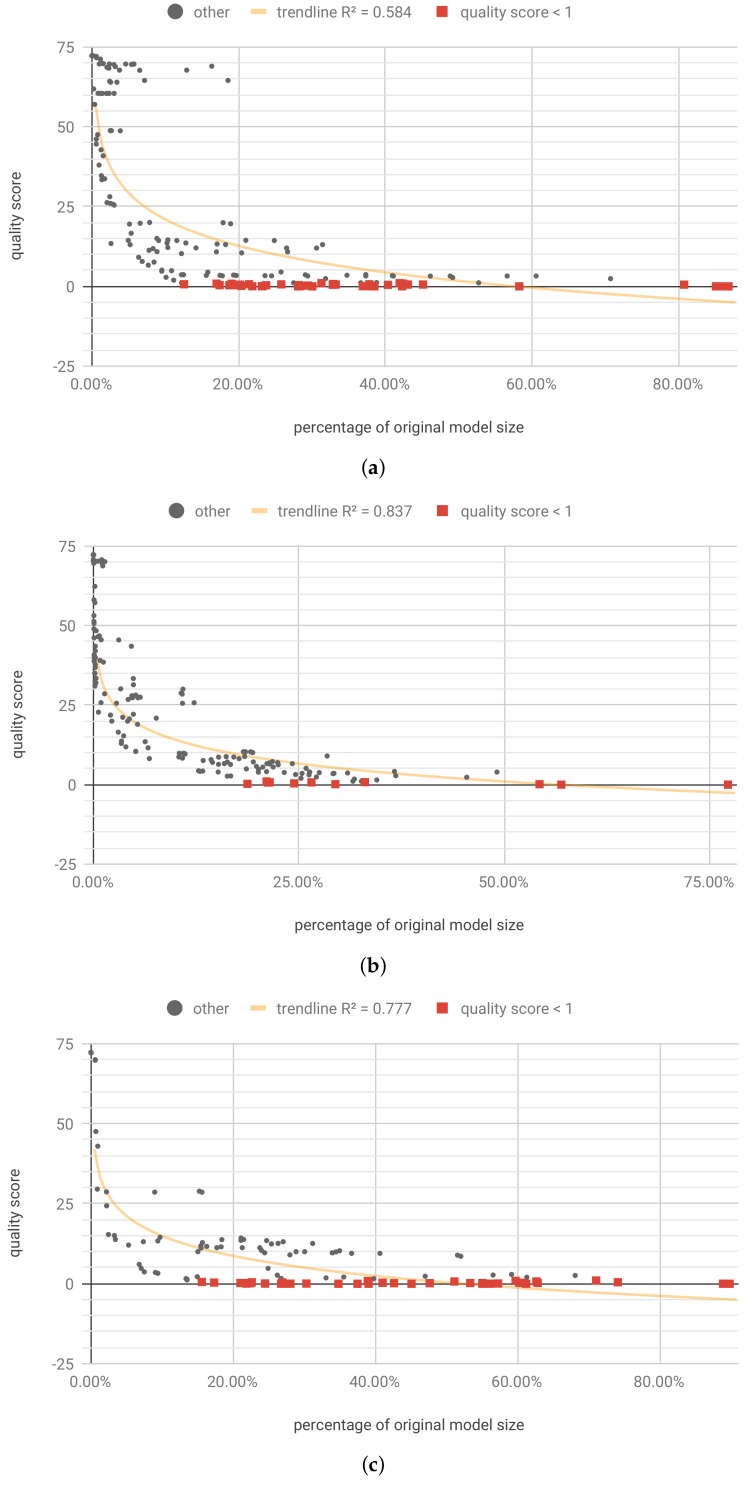
[PM2.5] Unique first Pareto front results. (**a**) Flow A: per-model pruning and quantization. trendline(x)=−6.6−12lnx; (**b**) flow B: individualized pruning and quantization. trendline(x)=−4.65−8.13lnx; (**c**) flow C-A: pruning and quantization with single retraining epoch. trendline(x)=−5.87−9.04lnx.

Optimization resulted in 165 unique Pareto optimal solutions, 39 of which retained the required qualityscore<1. For selected of those solutions, the hardware resources consumption and latency were simulated and presented in Table 2. The full results are shown in Table A4 (Appendix E) and Figure 11a. In terms of percentage of original model size, the best result for this dataset is comparable to the one achieved for International Airline Passengers (Section 3.1.2), but the Pareto front is much larger.

#### 3.2.3. Individualized Pruning and Quantization

The individualized pruning and quantization optimization (flow B) was run for 10,800 steps. It resulted in 332 unique Pareto solutions, 11 of which retained the required quality. The results are presented in Table A5 (Appendix E) and Figure 11b.

In case of this dataset, the individualized pruning and quantization did not lead to as good results as applying the flow A. This inability to at least match the best per-model outcomes may indicate that the selected number of optimization steps was too low for the number of optimized parameters.

#### 3.2.4. Pruning and Quantization with Retraining

For optimization with retraining, we have limited the range of the optimized parameters to the number of bits ∈[4,31] and pruning fraction ∈[0,0.5). Optimization was run for 3600 steps and resulted in 111 unique Pareto optimal solutions, 40 of which retained the required quality score (<1). The results are presented in Table A6 (Appendix E) and Figure 11c. Adding the retraining operation yielded solutions with the slightly improved quality score; however, contrary to the example presented in Section 3.1.4, it does not seem to affect the model compression limits.

### 3.3. Superconducting Magnets

This case study was based on the experiments conducted in [17], which was focused on exploring the influence of history length and various data quantization schemes on the models’ performance. The data we used came from the PM database of CERN LHC, representing the state of the superconducting magnets. Four data channels were used: U_DIFF_ (total voltage), U_RES_ (resistive voltage), I_DCCT_ (current measured using Hall sensor), and I_DIDT_ (time derivative of the electric current). Anomalies were marked using QUENCHTIME field as a start and until the end of series. Overall, ≈26% of used samples were marked as anomalous, which allowed using the accuracy (see Equation (A15) in Appendix C) as one of the quality measures.

The currently used system can detect an anomaly (a quench) with a latency of ≈10ms. Such a low latency is needed to start the shutdown procedure and safely discharge the magnet, without it being damaged. This system, however, requires manual adjustments for each magnet type and needs to be reworked for new devices that will be introduced in high-luminosity (HL) LHC phase. Our recent work concentrates on proposing an alternative solution, based on RNNs, that could become a part of the new protection device [16,17]. In order to meet the hard real-time requirements, the RNN-based model needs to be mapped to hardware, more specifically the FPGA. Also, the FPGA reconfigurability is a vital trait due to the necessary modifications caused by the change of the data’ parameters during the operational period as a result of normal working conditions and the elements degradation. Consequently, the model should be updated regularly with a newly trained version to maintain the highest performance.

While the work on designing the exact solution that will ensure the required quality is still ongoing, the research has reached the stage at which the actual hardware results are needed. The mapping flow was tested using a Keras model comprised of two LSTM layers, first with 64 cells and second with 32 cells, and a two-unit dense layer (for one-hot encoding). Total number of model parameters can be seen in Table 3.

#### 3.3.1. Initial Conditions Analysis

The tested model comprises of two LSTM layers and a single dense layer. The exact tensors sizes for the used architecture can be seen in Table 3. Equation (1) was used along with the data in Table 3 to compute the critical amount of memory size, which is essential to proceed with the proposed procedures. The calculations are as follows:

(19) memory=(17664+12416+66)∗1bit∗0.05≈1507bit≈188B.

We also directly mapped the model to using 32-bit fixed-point precision without compression and it turned out that it exceeded available resources on the target FPGA, Xilinx Zynq UltraScale + MPSoC XCZU15EG. This result led us to the conclusion that the proposed methodology is essential in order to map the model to the FPGA.

#### 3.3.2. Pruning/Quantization Optimization

To speed up the optimization process (done according to flow A), we have extracted the testing subset containing ≈8% of original samples. The subset size was selected based on the incremental dataset boosting method. At first, mean and variance were calculated for a single batch with size = 16,384). Then, the dataset was expanded by another batch, with mean and variance calculated again. If they changed more than a selected threshold (in our case 1%) when compared to the previous values, the dataset was expanded by a single batch again. In the end, the quality measures for the subset were compared with original ones (as seen in Table 4), and similarity confirmed.

**Figure 12:**
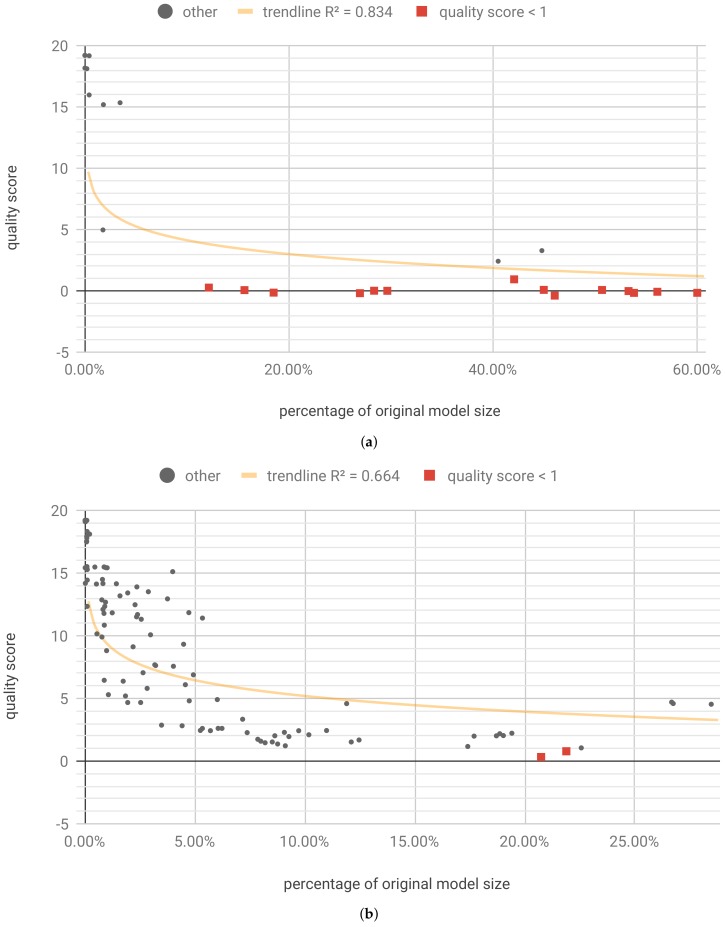
[LHC] Unique first Pareto front results for model trained on continuous data. (**a**) Flow A: per-model pruning and quantization. trendline(x)=0.364−1.63lnx; (**b**) flow B: individualized pruning and quantization. trendline(x)=1.02−1.81lnx.

Optimization was run for 3600 steps, and resulted in 34 unique Pareto optimal solutions (Figure 12a), 14 of which retained the required accuracy (drop <1p.p.). For those solutions, the quality score was validated using full test set and the hardware resources consumption and latency were simulated.

The simulation results are presented in Table 5. Different variants of quantization procedure were used along with different bit-width. Many of the solutions exceeded the resources’ capacity of the FPGA chip used for the experiments. However, the results which were obtained show that regardless of the procedure, the best 5 bit to 6 bit quantization leads to negligible accuracy drop and the best processing latency of a single chunk at the level of 210 ns.

We also run a short experiment with combining the *sparsity_score* and *used_bits_score* into a single loss value, it, however, yielded inferior results (out of 2400 optimization steps it generated 18 unique Pareto optimal solutions, only two of which retained the required accuracy).

#### 3.3.3. Individualized Pruning and Quantization

The optimization, with pruning and quantization parameters separate for each of the *W*, *U* and *b* tensors, ran for 16,200 steps. It resulted in 110 Pareto solutions (Figure 12b), but only two of those retained the required accuracy (drop <1p.p.), and further 12 results had drop <2p.p. The best results, presented in Table A7 (Appendix E), are slightly worse than the solutions found when applying flow A. This, in conjunction with the small number of results retaining the required quality may indicate, that the selected number of optimization steps was too low.

#### 3.3.4. Pruning and Quantization with Retraining

Pruning and retraining can be considered as one of the most flexible compression schemes since it enables weights shaping after each iteration step. However, it is worth noting that optimization with retraining can also be very time consuming, e.g., in our case a single pruning-quantization-retraining-pruning-quantization step took ≈23min, so the optimization running for 3600 steps was estimated to take nearly two months. Therefore, we decided to apply the retraining procedure only in conjunction with previously optimized parameters. The results are presented in Table 5 (in parentheses). For retraining, the full dataset was used, and in only in a few cases, the performance (accuracy) of the model improved. Based on those experiments, it can be seen that using the predefined pruning and quantization parameters, even previously optimized, does not guarantee a good starting point for the retraining.

#### 3.3.5. Data Bucketization

**Figure 13:**
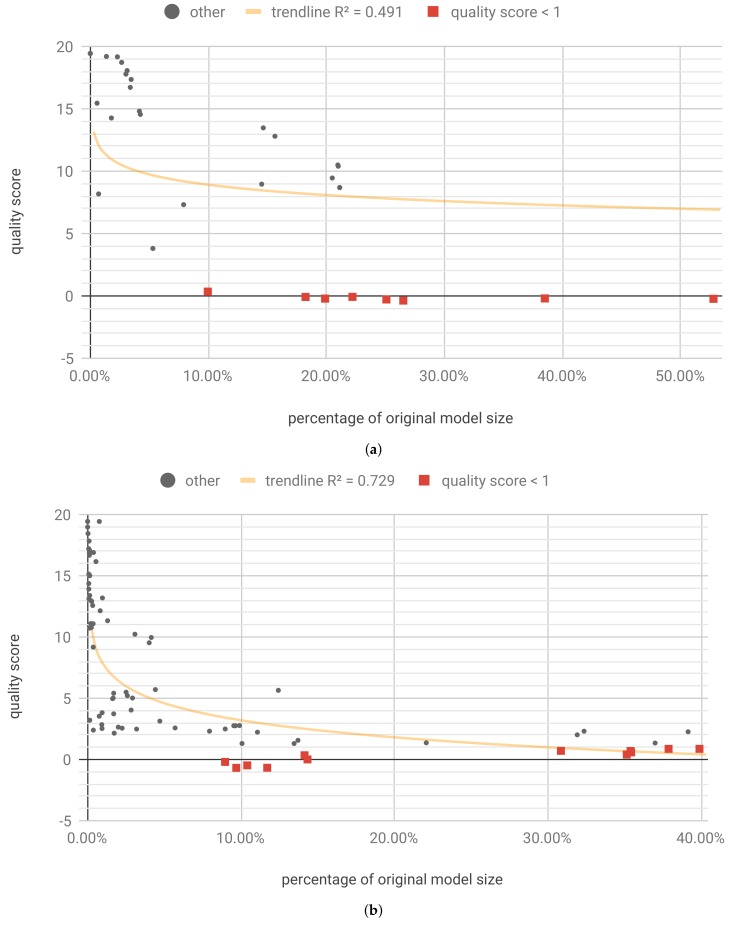
[LHC] Unique first Pareto front results for model trained on bucketized data. (**a**) Flow D-A: per-model pruning and quantization. trendline(x)=6.16−1.19lnx; (**b**) Flow D-B: individualized pruning and quantization. trendline(x)=−1.42−2.01lnx.

We also ran the experiments for flow D, using the recursive_adaptive data bucketization algorithm. It maps the input space to a fixed number of bins in such a way that all the resulting, uneven bins have similar cardinality if possible. For more details, please see Section 2.1 and Appendix A of [17], and Section 4.2 of [16]. The tested model structure was the same (two LSTM layers, with 64 and 32 cells, respectively, and a two-unit dense layer), which allowed us to focus on the differences caused by the data bucketization itself. The training on bucketized data yielded results on par with the original dataset (see Table 4). Also, in this case, for optimization, a test subset of ≈8% original size was used. Optimization was run for 3600 steps, and resulted in 42 unique Pareto optimal solutions, eight of which retained the required accuracy (Figure 13a).

The best results, presented in Table 6, show that data bucketization can be beneficial—the latency of the model is in a similar range as in Table 5 with quite low resources consumption.

Applying individualized pruning and quantization, with optimization running for 16,200 steps, yielded 12 results meeting the accuracy criterion (Figure 13b). The best results are shown in Table A8 (Appendix E). It is worth noting that individualized pruning and quantization (flow B) along with data bucketization, in this case, yield better results in terms model size after compression.

### 3.4. DL2HDL Comparison with Other Frameworks

To our best knowledge, the only high-level tool that can achieve comparable latency for neural network inference in FPGA is hls4ml [10]. This tool was designed to work in particle physics experiments, where response time is crucial. It provides similar functionality using a different approach.

The main difference between our flow and the hls4ml processing architecture is the number of procedure’s stages. In hls4ml, in the first stage, the model is created in one of the high-level DL frameworks, such as Keras or PyTorch, then it is mapped to Vivado HLS, and in the last step, the FPGA RTL structure is generated by the synthesis and implementation tool. Our DL2HDL tool skips the middle step and provides mapping directly from Python to HDL. It is worth noting that the number of conversion steps affects the flexibility of the tool and may a source of potential mapping challenges. Thus, we decided to simplify the flow when designing the DL2HDL.

Since at the time of this article submission the hls4ml support for recurrent neural networks was not yet available, we were not able to test it on network architectures examined in this work. However, it was possible to test the DL2HDL using the very similar network to the one presented in [10].

The tested architecture was a four-layer fully connected network, with ReLU as activation of the first three layers and softmax as output activation. Although we believe that softmax activation can be omitted in this application, we included it for a fair comparison. We used the same 16 bit wide fixed-point representation and pruned the network to the same number of parameters (1338). After HDL generation, the implementation was performed using Vivado software, for Xilinx Kintex Ultrascale xcku115-flvb2104-2-i with a target frequency of 200 MHz, as in the original experiment. Weights were randomly generated with uniform distribution.

Implementation results are summarized in Table 7. DPS usage and latency are on a similar level, although our implementation is slightly faster. Logic utilization, however, is significantly lower when using DL2HDL.

We also compared our flow with the LeFlow [8] tool-kit. In the setup, we used the results for dense_a and dense_b reported in the paper. They are single dense layers including bias and ReLU activation with a single input and 8 and 64 outputs respectively. Using DL2HDL, we have generated layers with the same parameters. Results are summarized in Table 8. Resources utilization is not fully comparable as authors in [8] performed tests on Altera Stratix IV EP4SGX290NF45C3, while our tests were done on Xilinx xczu15egffvc900-1. However, thanks to the full unroll of operations and weights hardcoded in logic, DL2HDL required significantly less time to infer through the layers.

## 4. Discussion

This paper proposes both methodology and its implementation tools. We did experiments to validate them, which revealed a series of application aspects related to the effectiveness of the steps and their recommended execution. We showed that the presented methodology and the tool could be successfully applied to IoT mapping scenarios for latency and resources consumption estimation and validation.

We proposed a method to estimate model size in Equation (15), which assumes that linear quantization is employed. Despite its simplicity, the method allows picking candidate results among the Pareto front solutions for further processing e.g., mapping to hardware.

Figure 10, Figure 11, Figure 12 and Figure 13 present the optimization results as a function of the estimated model size. Moving towards the origin of the coordinate system leads to a growth of the quality score (degradation of the model performance). In every model, there exist a core amount of resources which captures the essence of the underlying processed modeled by the neural network [37]. Once the critical resources are removed by pruning and quantization, the performance of the model starts to decline very fast. This degradation usually occurs around 5% to 10% of the original model size. We have tested several trendline models and discovered that, in general, the logarithmic trendline yielded good results in terms of R^2^ metric.

The per-model scheme (flow A) seems to yield satisfactory results in most of the tested cases. The solutions yielded by flow B seem to be inferior to the per-model approach while reaching them required more computation time. We believe, however, that the limiting factor was the selected number of optimization steps, calculated according to Equation (13). The individualized optimization, running for a sufficient number of steps, should be able to reach at least the same performance as the per-model one [37]. Also, when distributed implementation is at the premium, it may turn out that the individualized approach may allow for better optimization from the perspective of each computing node in the IoT network.

In the presented experiments, we limited our retraining setup to a single epoch of training, which does not take a full adventure of this procedure. Training for multiple epochs, especially taking into account loss function change to adjust learning rate, may lead to better results. It is also expected that applying regularization techniques (such as weight decay) during training or retraining process may lead to even better compression results [13,14]. While our setup accounts for this option in general, it should be extended with a series of meta-parameters to use retraining for multiple epochs properly. Careful selection of parameters, such as the number of epochs, learning rate adjustment schedule and dataset size, is vital because retraining, especially with a full dataset is very time consuming and may be counter-productive if not carefully implemented. Using a reduced dataset, possibly newly selected in each epoch, and introduction of sensitivity list for the model’s layers may significantly boost the effects of retraining procedure.

One of the possible compression flows for big models, not discussed in this work, is based on the idea of incremental pruning and quantization. In this flow, introducing the sensitivity list allows picking fragments of the model, which are pruning prone and should be addressed as first. Especially in conjunction with retraining, the sensitivity list should be updated quite often, since the profile of the model changes as the training progress. The process of refreshing of the sensitivity list by itself is also computationally demanding. Therefore, it should also be deliberately adjusted and implemented [38].

We applied bucketization in one of the experiments and achieved comparable results to using continuous data. Though, we noticed that the individualized scheme yielded better results with bucketization. Additionally, in sensors networks with stringent energy and latency budget, data transmission cost plays a critical role in the system. Bucketization considered as a form of a compression scheme, enables dynamic data stream modulation with graceful performance degradation.

Comparison of unstructured pruning applied to FPGA and GPGPU/CPU is quite challenging, since the latter platforms cannot directly benefit from DL models compression unless a dedicated data structuring scheme is implemented [39,40,41,42]. This scheme is essential to take advantage of sparse vector-matrix and matrix-matrix multiplication operations, which are much more efficient than their dense counterparts, provided the data is prepared properly. In contrast, FPGA-based solutions directly benefit from unstructured pruning and quantization as the underlying internal structure of the platforms is adaptable and reconfigurable. For illustrative purposes, in Table 9, we presented the comparison between inference times on different platforms.

There is a range of modifications which may be introduced to the proposed optimization routine based on MO-CMA-ES. One of the most impactful can be the improvement of the loss function, which reformulated may affect the performance of the optimization process. At the early stage of our experiments we made a simple, unsuccessful attempt to merge the sparsity score and used bits score into a single score; however, we believe that a more sophisticated combination may yield better results.

Multi-objective optimization is a quite complex operation; thus it is not easy to come up with a stopping criterion which on hand would allow the process to converge, and on the other hand, would not unnecessarily prolong the process. In this work, we chose to run the optimization for the arbitrary number of steps, which, as mentioned, turned out to be a limiting factor. Using another form of the stopping criterion could potentially lead to better results. An alternative could be developing a different way calculating the maximum number of steps.

An interesting observation in terms of FPGA resources consumption is irregularity of its distribution. For instance, in the course of the experiments, we found out that there is a component in the model, which contributed vastly to the overall resources consumption. Because of the non-hierarchical structure of the generated HDL code using MyHDL, it was hard to determine which section it was. Fortunately, after some examination we determined tanh activation function as the culprit (see Appendix D). As the next step, the module was re-implemented with tanh as a linear approximation of the original function rather than Taylor function expansion. This change significantly reduced the overall resources consumption (see Table A1). Not only it decreased the number of DPS blocks within FPGA, but also changed a profile of available resources to the other submodules within the implemented model, making it more balanced.

DL2HDL allows mapping DL models with arbitrary precision and sparsity to FPGA. It is a part of the whole presented flow. The design choices we made during the tool development have allowed reducing recurrent network inference latency to the scale of tens to hundreds of nanoseconds. The standard interface between individual elements facilitates future development and refactoring of network layers.

As future work, we plan to investigate all the described improvements of the optimization flow, as well as to conduct an in-depth analysis of basic neural operators such as exp and tanh in the context of model pruning and quantization.

## 5. Conclusions

The proposed mapping methodology and tools may be used in FPGA-based IoT devices for model compression. It can also be employed for distributed systems since its application in every node is independent and also can be ported across processing elements within the sensors network. The methodology also proposes steps to be taken in order to assess potential resources consumption of the system. It may be incorporated as a backbone element in a larger environment for a generation or ad-hoc reconfiguration of a network infrastructure.

Our DL2HDL tool matches or supersedes the state-of-the-art solutions in terms of the achieved latency, but the architecture is more uniform and allows mapping directly from Python to HDL. Our solution generates HDL models with three orders of magnitude lower than LeFlow [8] and comparable to hls4ml [10]. For the LSTM network composed of four cells, it reached as low latency as 27 ns with only 143 LUTs consumed of Xilinx Zynq UltraScale+ MPSoC XCZU15EG while for a three-layer network with 32 and 16 LSTM cells and a two-unit dense layer on top it achieved the latency of 210 ns.

## Figures and Tables

**Table 1:** [IAP] Results for pruned and quantized model. Percentages in the ‘#LUT’ and ‘#DSP’ refer to the overall resources available on the chip.

Pruning		Quantization		*Quality Score*	Sparsity (%)	% of Original Size; Equation (15)	#LUT	#DSP	Latency (ns)
Method	Fraction	Method	Bits						
simple	0	thq	4	−27.08	0	12.50	143 (0.04%)	0 (0%)	27
hist_amount	0.1916	linear	5	−8.52	23.08	12.38	427 (0.13%)	0 (0%)	37
hist_amount	0.2379	thq	5	−2.54	23.32	12.22	403 (0.12%)	0 (0%)	36
simple	0.1286	thq	13	0.89	27.64	30.57	1634 (0.48%)	85 (2.41%)	111

**Table 2:** [PM2.5] Selected results for pruned and quantized model. Percentages in the ‘#LUT’ and ‘#DSP’ refer to the overall resources available on the chip. Full results are available in Table A4.

Pruning		Quantization		*Quality Score*	Sparsity (%)	% of Original Size; Equation (15)	#LUT	#DSP	Latency (ns)
Method	Fraction	Method	Bits						
simple	0.0312	linear	15	−0.01	13.46	42.30	21,125 (6.19%)	1559 (44.19%)	110
simple	0.0380	log_linear	21	−0.01	14.75	58.27	36,393 (10.66%)	1634 (46.32%)	132
simple	0.0237	linear	6	0.34	10.03	17.42	5422 (1.59%)	0 (0%)	54
hist	0.0924	linear	11	0.37	19.22	28.26	18,886 (5.53%)	655 (18.57%)	101
simple	0.0596	linear	31	0.53	20.73	80.74	46,441 (13.61%)	3178 (90.08%)	184
simple	0.0675	log_linear	17	0.55	21.33	43.08	23,024 (6.75%)	1499 (42.49%)	113
simple	0.0862	linear	19	0.56	26.30	45.14	24,703 (7.24%)	1424 (40.36%)	120
simple	0.0625	linear	10	0.61	20.91	25.83	27,095 (7.94%)	0 (0%)	84
simple	0.0874	log_linear	16	0.63	26.40	37.83	20,003 (5.86%)	1382 (39.17%)	111
hist	0.2085	thq	9	0.65	22.65	21.43	17,697 (5.19%)	0 (0%)	70
hist	0.1185	linear	5	0.65	19.70	12.57	972 (0.28%)	0 (0%)	57
hist	0.2323	linear	14	0.73	22.91	32.91	19,383 (5.68%)	1316 (37.3%)	110
hist	0.2074	linear	8	0.80	22.65	19.05	12,411 (3.64%)	0 (0%)	69
simple	0.0786	linear	7	0.83	24.93	17.00	7751 (2.27%)	0 (0%)	60
simple	0.0908	linear	18	0.99	28.50	41.95	22,591 (6.62%)	1375 (38.97%)	119
simple	0.0811	linear	13	0.99	25.13	31.30	18,170 (5.32%)	1166 (33.05%)	112

**Table 3:** [LHC] Sizes of tensors containing the model weights.

LSTM_1_				LSTM_2_				Dense			Total
$W_{1}$	$U_{1}$	$b_{1}$	Total	$W_{2}$	$U_{2}$	$b_{2}$	Total	$W_{D}$	$b_{D}$	Total	
1024	16,384	256	17,664	8192	4096	128	12,416	64	2	66	30,146

**Table 4:** [LHC] Models’ predictions quality depending on test set size. For quality measures definitions see Appendix C.

Data	Bucketization	Accuracy	F_1_ Score	F_2_ Score	Precision	Recall	Total Samples	Anomalous Data (%)
all	no	0.8604	0.7892	0.7913	0.7857	0.7927	499020	32.96
	yes	0.8634	0.7869	0.7738	0.8098	0.7654		
subset	no	0.8586	0.7888	0.7910	0.7853	0.7924	40960	33.34
	yes	0.8609	0.7857	0.7733	0.8074	0.7652		

**Table 5:** [LHC] Results for pruned and quantized model trained (and retrained) on continuous data. In ‘*quality score*’ and ‘sparsity’ columns, values in parentheses indicate results obtained when using model retraining (see Section 3.3.4), with results better than with no retraining marked in bold. Pauses indicate results, for which the resources consumption exceeded the XCZU15EG FPGA capacity. Percentages in the ‘#LUT’ and ‘#DSP’ refer to the overall resources available on the chip.

Pruning		Quantization		*Quality Score*		Sparsity (%)	% of Original Size; Equation (15)	#LUT	#DSP	Latency (ns)
Method	Fraction	Method	Bits	Optimization	Full					
hist_amount	0.0816	linear	16	−0.3955 (−0.0293)	−0.4058	8.10 (8.10)	46.01	–	–	–
hist_amount	0.3365	minmax	13	−0.1953 (0.8179)	−0.1645	33.71 (33.54)	26.93	–	–	–
hist_amount	0.3385	linear	26	−0.1709 (**−0.4004**)	−0.1495	33.76 (33.76)	53.77	–	–	–
hist_amount	0.3392	linear	29	−0.1660 (−0.0269)	−0.1479	33.76 (33.76)	59.97	–	–	–
simple	0.0035	minmax	6	−0.1514 (**−0.1831**)	−0.2222	2.13 (**4.28**)	18.48	41,991 (12.30%)	4 (0.11%)	263
hist_amount	0.4199	linear	31	−0.0806 (51.8018)	−0.1741	41.86 (41.86)	56.06	–	–	–
hist_amount	0.3449	linear	26	−0.0244 (0.9424)	0.0573	34.71 (34.71)	53.24	–	–	–
hist_amount	0.1391	linear	11	−0.0024 (**−0.2466**)	0.0142	13.96 (13.96)	29.61	260,116 (76.22%)	1729 (49.01%)	477
hist_amount	0.1764	linear	11	0.0049 (1.0889)	0.0465	17.25 (**17.42**)	28.32	253,541 (74.29%)	1711 (48.50%)	443
simple	0	thq	5	0.0537 (0.0537)	0.1148	0 (0)	15.63	13,694 (4.01%)	4 (0.11%)	210
hist_amount	0.3499	linear	25	0.0659 (0.1050)	0.1170	35.08 (35.08)	50.64	–	–	–
hist_amount	0.3474	linear	22	0.0732 (**0.0122**)	0.1345	34.77 (34.77)	44.94	–	–	–
hist_amount	0.3541	log_minmax	6	0.2588 (**0.1929**)	0.2950	35.80 (35.80)	12.14	38,147 (11.18%)	4 (0.11%)	253
hist_amount	0.4812	linear	26	0.9326 (51.4990)	0.9683	47.88 (41.86)	42.03	–	–	–

**Table 6:** [LHC] Results for a pruned and quantized model trained on bucketized data. Pauses indicate results, for which the resources consumption exceeded the XCZU15EG Field-programmable gate array (FPGA) capacity. Percentages in the ‘#LUT’ and ‘#DSP’ refer to the overall resources available on the chip.

Pruning		Quantization		*Quality Score*		Sparsity (%)	% of Original Size; Equation (15)	#LUT	#DSP	Latency (ns)
Method	Fraction	Method	Bits	Optimization	Full					
hist_amount	0.2266	linear	11	−0.3735	−0.2932	22.50	26.51	243,216 (71.27%)	1693 (47.99%)	443
hist_amount	0.1984	linear	10	−0.2856	−0.2058	19.42	25.08	199,905 (58.58%)	292 (8.28%)	376
hist_amount	0.2318	log_minmax	22	−0.2344	−0.1918	22.64	52.77	–	–	–
hist_amount	0.0909	thq	7	−0.2173	−0.1132	9.01	19.89	68,903 (20.19%)	6 (0.17%)	354
hist_amount	0.2314	linear	16	−0.2051	−0.1788	22.55	38.49	–	–	–
hist_amount	0.1665	linear	7	−0.0854	−0.0663	16.63	18.23	66,714 (19.55%)	6 (0.17%)	344
hist_amount	0.2090	linear	9	−0.0781	−0.0419	21.30	22.20	140,061 (41.40%)	298 (8.45%)	376
simple	0.0429	minmax	4	0.3394	0.3401	31.42	9.95	9067 (2.66%)	9299 (1.36%)	210

**Table 7:** Comparison with hls4ml framework, with percentage difference to original experiment [10].

	hls4ml [10]	DL2HDL	
#DSP48E	954	1069	(112.05%)
#LUT + #FF	88,797	38,146	(42.96%)
latency (ns)	75	70	(93.33%)

**Table 8:** Comparison with the LeFlow framework.

	LeFlow [8]			DL2HDL			
	#LE	MemB	Latency (ns)	#LUT	#FF	#DSP	Latency (ns)
dense_a	1743	1056	1421	110	300	16	4.83
dense_b	1749	8224	10,343	4848	2154	124	5.15

**Table 9:** Inference times for a single sample on various devices. The general-purpose graphics processing unit (GPGPU) and central processing unit (CPU) experiments were done with uncompressed models, since unstructured pruning and quantization does not significantly affect the performance. For FPGA, the best per-model pruning and quantization results were used.

	GPGPU (ns)	CPU (ns)	FPGA (ns)
	Nvidia GeForce GT 730M	Intel i5-4210M	Xilinx Zynq UltraScale+ MPSoC XCZU15EG
**[IAP]**	0.72 × 10^6^	0.81 × 10^6^	27
**[PM2.5]**	5.33 × 10^6^	7.22 × 10^6^	54
**[LHC]**	39.5 × 10^6^	42.19 × 10^6^	210

**Figure A1:**
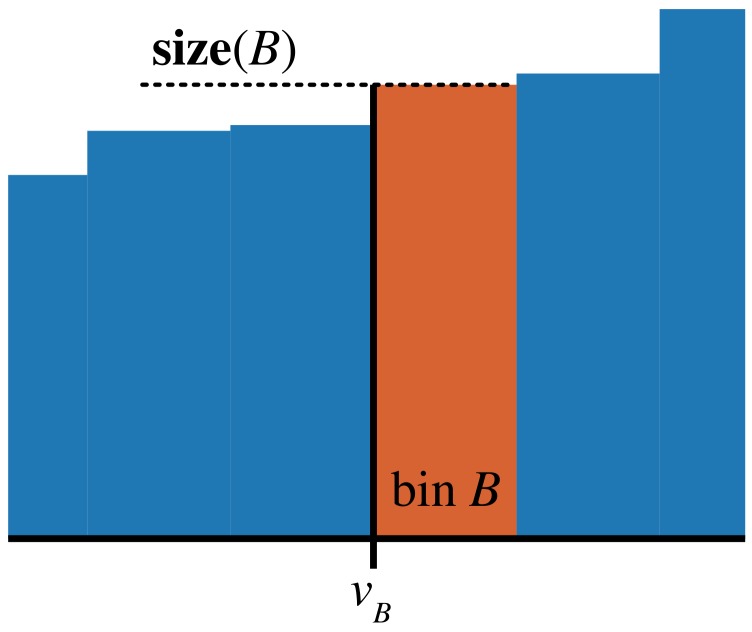
The ‘hist’ method concepts visualized using a histogram fragment.

**Figure A2:**
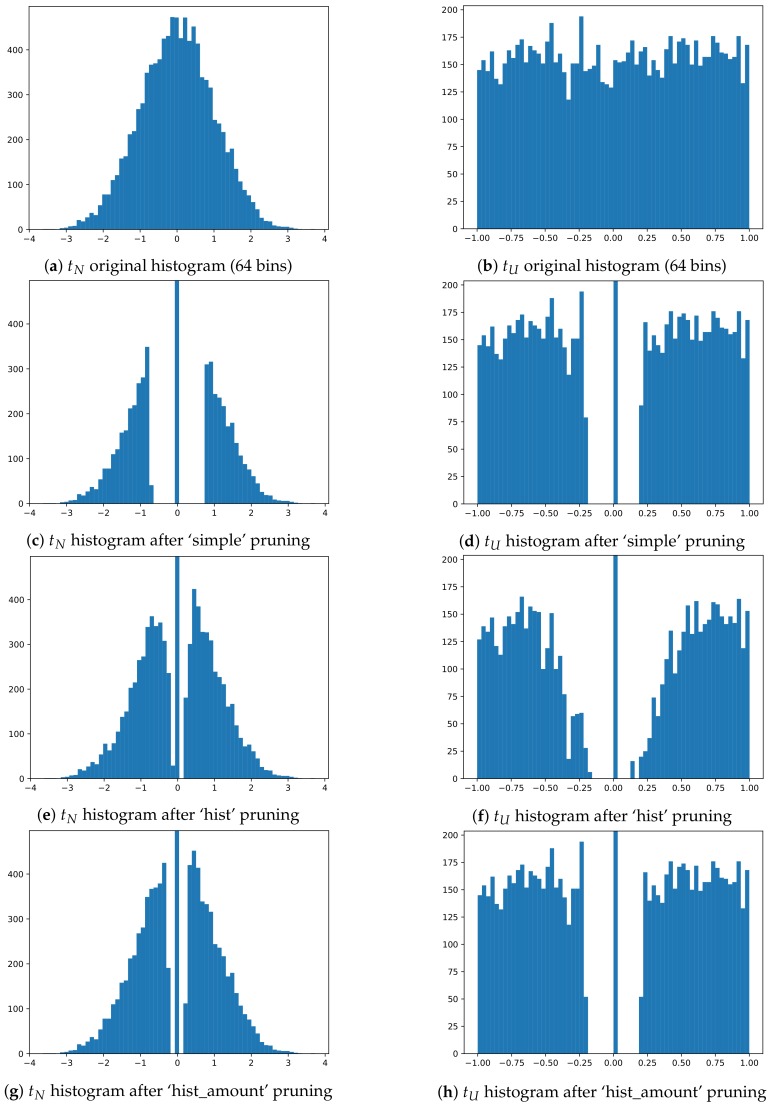
Application of different pruning methods for two tensors: $t_{N}$—sampled from normal distribution, and $t_{U}$—sampled from uniform distribution and scaled to (−1,1) range. The tensors length was 10^4^. For each method, the pruning fraction =0.2.

**Figure A3:**
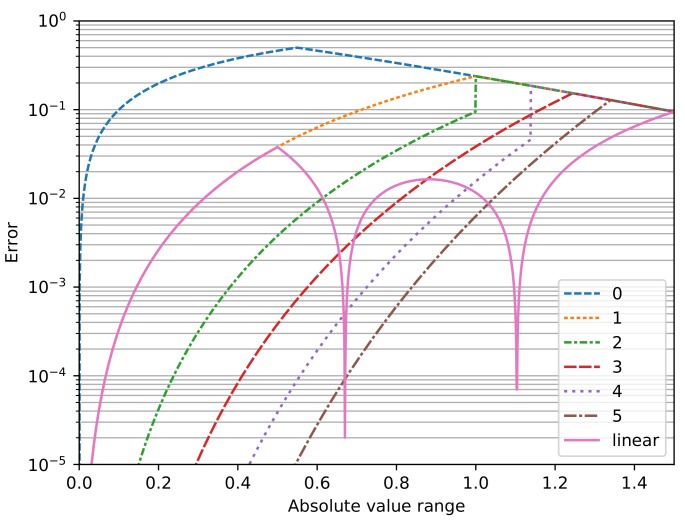
Tanh error as function of *x*. Polynomial degree is indicated with different colors.

## Supplementary Materials

The developed time series analysis software ‘analysta’ is available online at https://bitbucket.org/maciekwielgosz/anomaly_detection. The DL2HDL mapping tool is available at https://bitbucket.org/maciekwielgoszteam/dl2hdl.

## Abbreviations

The following abbreviations are used in this manuscript:

ADC	analog-to-digital converter
API	application programming interface
ASIC	application-specific integrated circuit
CERN	European Organization for Nuclear Research
CMA-ES	covariance matrix adaptation evolution strategy
CNN	convolutional neural network
CPU	central processing unit
DL	deep learning
ES	evolution strategy
FPGA	field-programmable gate array
GA	genetic algorithm
GPGPU	general-purpose graphics processing unit
HDL	hardware description language
HL	high-luminosity
HLL	high-level language
IoT	internet of things
LHC	Large Hadron Collider
LSTM	long short-term memory
MO-CMA-ES	multi-objective covariance matrix adaptation evolution strategy
p.p.	percentage point
PM	post mortem
RMSE	root-mean-square error
RNN	recurrent neural network
TSA	time series analysis

## Appendix A. Pruning Methods

In experiments, three pruning methods were considered: simple, hist, and hist_amount. In the following equations, w∈W is used to denote the original value from the layer’s weights *W*.

The ‘simple’ pruning method sets all weights with an absolute value lower than a fraction $f_{\mathrm{simple}}$ of maximal absolute weights’ value to zero:

(A1) $$\mathrm{simple}\left(w\right)=\left\{\begin{array}{cc} 0 & \mathrm{if}\;|w|<f_{\mathrm{simple}}\;\cdot \;\max\left(|\min(W)|,|\max(W)|\right),\\ w & \mathrm{otherwise}. \end{array}\right.$$

In the ‘hist’ method, the weights’ values are divided into $2^{12}$ equal bins. For each bin *B* with cardinality size(B) and lower bin edge value $v_{B}$ (see Figure A1), the score $s_{B}$ is calculated:

(A2) $$s_{B}=\sqrt[{3}]{{\left(\mathrm{size}\left(B\right)\;\cdot \;v_{B}\right)}^{8}}.$$

Then, the average score value $\bar{s}$ is found and used to calculate a threshold, expressed as a fraction $f_{\mathrm{hist}}$ of the average score. The threshold is then compared with each bin’s individual score $s_{B}$. Finally, all the weights falling into the bins with scores below the threshold are set to zero:

(A3) $$\mathrm{hist}\left(w\right)=\left\{\begin{array}{cc} 0 & \mathrm{if}\;w\in B:s_{B}<f_{\mathrm{hist}}\;\cdot \;\bar{s},\\ w & \mathrm{otherwise}. \end{array}\right.$$

The ‘hist_amount’ method zeroes weights until a target fraction $f_{\mathrm{hist}\_\mathrm{amount}}$ of layer weights *W* is changed (see Algorithm A1). The weights are divided into equal $2^{10}$ bins. The procedure starts with bins containing weights with the lowest absolute value, i.e., the absolute value of lower edge of bin $B_{0}$ is smaller than the absolute value of the lower edge of bin $B_{1}$, etc.

**Algorithm A1** hist_amount pruning method	
*pruned* ← 0	▹ set the number of pruned weights to 0
*i* ← 0	▹ used for bins indexing
**while** *pruned* < *f_hist_amount_* · **size** (*W*) **do**	▹ *pruned* is lower than the threshold
**for all** *w* ∈ *B_i_* **do**	▹ set all the weights in the bin B_i_ to 0
*w* ← 0	
**end for**	
*pruned* ← *pruned* + **size** (*B_i_*)	▹ update the pruned weights amount
*i* ← *i* + 1	
**end while**	

As can be seen in Figure A2, the effect of specific pruning method application depends on the weights distribution. For normally-distributed data, ‘hist’ and ‘hist_amount’ yield similar results, while for uniformly-distributed the similarities exist between ‘simple’ and ‘hist_amount’.

## Appendix B. Quantization Methods

For the presented experiments, we adopted common quantization methods. In the following equations, w∈W is used to denote the original value from layer’s weights *W*, and **bits** is the target number of bits. In the implementation, when taking the logarithm, a tiny constant is added to prevent errors related to taking the logarithm of zero.

It is worth emphasizing that using non-linear quantization procedures, such as presented in Equations (A10)–(A12), requires in-hardware mapping schemes. These schemes are supposed to encode unequally distributed quantized values to equally-spaced memory locations. The mapping is a LUT operation, which outputs quantized values for corresponding input data. In hardware FPGA implementation, it can be implemented by using BRAM or distributed RAM to store the mapping. Access to the memory should be fast with low latency since the mapping procedure will be used for all the operations which involve using quantized values. Fast access is essential since the internal arithmetic modules such as MACs units of multipliers work with evenly-distributed fixed-point numbers. Consequently, from a performance perspective, it is not recommended to keep the maps in an external memory such as SDRAM for which as access time is extended.

The linear quantization can be defined as follows:

(A4) $$\mathrm{linear}\left(w\right)=s_{\mathrm{linear}}\;\cdot \;\mathrm{clip}\left(\left\lfloor \frac{w}{s_{\mathrm{linear}}}+\frac{1}{2}\right\rfloor ,-2^{\mathrm{bits}-1},2^{\mathrm{bits}-1}-1\right),$$

where scaling factor $s_{\mathrm{linear}}$, the number of bits needed to store the integral part int_bits, and the clipping function clip(a,min,max) are defined as:

(A5) $$s_{\mathrm{linear}}=\frac{1}{2^{\mathrm{bits}-1-\mathrm{int}\_\mathrm{bits}}},$$

(A6) $$\mathrm{int}\_\mathrm{bits}=\left\lceil \log_{2}\max(W)\right\rceil ,$$

(A7) $$\mathrm{clip}\left(a,\min,\max\right)=\left\{\begin{array}{cc} \min & \mathrm{if}\;a<\min,\\ a & \mathrm{if}\;\min\leq a\leq \max,\\ \max & \mathrm{otherwise}. \end{array}\right.$$

Another quantization method is based on finding the extreme values in the set:

(A8) $$\mathrm{minmax}\left(w\right)=s_{\mathrm{minmax}}\;\cdot \;\left\lfloor \frac{w-\min(W)}{s_{\mathrm{minmax}}}+\frac{1}{2}\right\rfloor +\min\left(W\right),$$

with scaling factor $s_{\mathrm{minmax}}$:

(A9) $$s_{\mathrm{minmax}}=\frac{\max(W)-\min(W)}{2^{\mathrm{total}}-1}.$$

The ‘log’ versions of the above quantization methods can be described as:

(A10) $$\log\_\mathrm{linear}\left(w\right)=\mathrm{sgn}\left(w\right)\;\cdot \;e^{\mathrm{linear}\left(\ln\left|w\right|\right)},\mathrm{and}$$

(A11) $$\log\_\mathrm{minmax}\left(w\right)=\mathrm{sgn}\left(w\right)\;\cdot \;e^{\mathrm{minmax}\left(\ln\left|w\right|\right)}.$$

The last tested method is based on the hyperbolic tangent:

(A12) $$\mathrm{thq}\left(w\right)=\mathrm{arctanh}\left(s_{\mathrm{thq}}\;\cdot \;\left\lfloor \frac{\tanh(w)+1}{s_{\mathrm{thq}}}+\frac{1}{2}\right\rfloor -1\right),$$

where scaling factor $s_{\mathrm{thq}}$:

(A13) $$s_{\mathrm{thq}}=\frac{2}{2^{\mathrm{total}}-1}.$$

## Appendix C. Quality Measures

The root-mean-square error used during experiments was calculated according to the formula:

(A14) $$\mathrm{RMSE}=\sqrt{\frac{1}{N}\sum_{i=1}^{N}{\left(y_{r}^{i}-y_{p}^{i}\right)}^{2}}$$

where $y_{r}^{i}$ and $y_{p}^{i}$ are a time series and its predicted counterpart, respectively, and *N* is a dataset cardinality.

Given the values *t* and *f* representing, respectively, an amount of correctly and incorrectly classified samples, the accuracy can be defined as in (A15):

(A15) $$\mathrm{accuracy}=\frac{t}{t+f}.$$

An F score is calculated using two helper metrics, a recall (A16), also called sensitivity, and a precision (A17):

(A16) $$\mathrm{recall}=\frac{\mathrm{tp}}{\mathrm{tp}+\mathrm{fn}},$$

(A17) $$\mathrm{precision}=\frac{\mathrm{tp}}{\mathrm{tp}+\mathrm{fp}},$$

where:

- tp—true positive—item correctly classified as an anomaly,
- fp—false positive—item incorrectly classified as an anomaly,
- fn—false negative—item incorrectly classified as a part of normal operation.

The β parameter controls the recall importance in relevance to the precision when calculating an F score:

(A18) $$F_{\beta }=\left(1+\beta^{2}\right)\;\cdot \;\frac{\mathrm{recall}\;\cdot \;\mathrm{precision}}{\mathrm{recall}+\beta^{2}\;\cdot \;\mathrm{precision}}.$$

During the experiments, two β values were used, 1 and 2, to show the impact of the recall on the final score.

For the trendline models in Figure 10, Figure 11, Figure 12 and Figure 13, the R^2^ (coefficient of determination) score was calculated according to the following formula:

(A19) $$R^{2}=1-\frac{\sum_{i=1}^{N}{\left|y_{r}^{i}-{\hat{y}}_{r}\right|}^{2}}{\sum_{i=1}^{N}{\left|y_{r}^{i}-y_{p}^{i}\right|}^{2}}$$

## Appendix D. Tanh Error

Figure A3 presents an error introduced by tanh polynomial approximation using Taylor series according to Equation (A20). The result is clipped to range −1 to 1. Additionally, to ensure that the function is monotonous, the result is set to the closest limit value after the point where monotonicity is broken, this effect is visible for even number of polynomial degree.

(A20) $$\tanh\left(x\right)=x-\frac{x^{3}}{3}+\frac{2x^{5}}{15}-\frac{17x^{7}}{315}+\frac{62x^{9}}{2835}-\ldots$$

It may be noticed that an increase of polynomial degree leads to more accurate approximation for higher values. It is worth noting that for normalized model weights, the values are close to zero. Therefore, using lower polynomial degree may be enough to obtain an accurate result. On the other hand, incorporating more complex approximation leads to the massive growth of the resources consumption, as presented in Table A1.

**Table A1:** Resources consumption for various tanh(x) approximation setups.

Implementation	Bits	LUT		FF		DSP	
		#	%	#	%	#	%
linear	32	6862	2.01	2062	0.30	0	0.00
series		369,210	108.18	34,117	5.00	3273	92.77
linear	16	2948	0.86	1038	0.15	0	0.00
series		26,765	7.84	9716	1.42	2080	58.96
linear	12	2212	0.65	782	0.11	0	0.00
series		16,818	4.93	7283	1.07	1088	30.84
linear	10	548	0.16	622	0.09	0	0.00
series		10,372	3.04	6067	0.89	672	19.05
linear	8	324	0.09	494	0.07	0	0.00
series		4516	1.32	2834	0.42	256	7.26
linear	6	147	0.04	398	0.06	0	0.00
series		1764	0.52	1362	0.20	256	7.26

As polynomial approximation requires a significant amount of resources, linear approximation was also implemented (Equation (A21)). Its accuracy is lower, however maximal error lies around 4–5 bit 0.5 LSB and resource requirements are substantially smaller.

(A21) $$\tanh\left(x\right)=\left\{\begin{array}{cc} -1 & x\leq -1.5\\ 0.25*x-0.25 & -1.5\leq x\leq -0.5\\ x & -0.5\leq x\leq 0.5\\ 0.25*x+0.25 & 0.5\leq x\leq 1.5\\ 1 & 1.5\leq x \end{array}\right.$$

## Appendix E. Detailed Optimization Results

This Appendix contains the detailed results for International Airline Passengers **[IAP]** (Table A2 and Table A3), Beijing PM_2.5_ Pollution **[PM2.5]** (Table A4, Table A5 and Table A6) and Superconducting Magnets **[LHC]** experiments (Table A7 and Table A8).

**Table A2:** [IAP] Results for individualized pruning and quantization.

Pruning Method	Pruning Fraction					Quantization Method	Quantization Bits					*Quality Score*	Sparsity (%)	% of Original Size; Equation (15)
	LSTM			Dense			LSTM			Dense				
	W	U	b	W	b		W	U	b	W	b			
hist_amount	0.4574	0.4644	0.6099	0.1076	0.9933	thq	20	1	2	4	2	−36.91	47.60	7.55
hist_amount	0.5720	0.8892	0.9388	0.2296	1	thq	2	9	1	4	9	−33.2	77.16	4.27
hist_amount	0.5410	0.958	1	0.1226	1	thq	2	10	1	4	8	−31.93	78.13	3.03
hist	0.4683	0	0.1861	0.8444	0.9846	thq	1	1	2	6	5	−31.45	11.54	13.46
hist_amount	0.5025	0.9349	1	0.2032	0.9981	thq	2	4	1	4	5	−30.46	77.64	2.32
simple	0	0.3598	0.8899	0	1	thq	1	1	1	4	4	−28.93	58.65	5.69
simple	0.0397	0.4340	0.8363	0	1	thq	1	1	1	4	5	−28.84	59.86	5.79
hist_amount	0.7180	0.9531	1	0.2103	0.9678	thq	2	10	1	5	9	−27.73	78.85	3.03
hist_amount	0.6365	1	0.9794	0.0655	1	thq	2	10	1	4	11	−27.02	79.33	2.04
hist_amount	0.5945	1	0.995	0.1000	1	thq	2	10	1	4	10	−27.01	79.09	2.17
hist_amount	0.6474	1	1	0.1369	1	thq	2	9	1	4	12	−26.98	79.33	1.92
hist_amount	0.5797	1	1	0.0952	1	thq	2	9	1	4	9	−26.95	79.09	2.04
hist_amount	0.5621	0.9864	1	0.1044	0.9983	thq	2	9	1	4	8	−26.91	78.85	2.17
hist_amount	0.5374	1	1	0.0651	1	thq	2	8	1	4	9	−26.85	78.85	2.04
hist_amount	0.7021	0.9906	1	0.2020	1	thq	2	16	1	5	10	−26.79	79.81	2.66
simple	0.0157	0.8750	0.9071	0.0746	0.0917	thq	2	1	1	4	4	−26.02	68.75	2.51
simple	0.0179	0.0046	1	0.0803	1	thq	1	1	1	4	3	−21.58	57.93	8.01
simple	0	0.0158	1	0.0010	0.9925	thq	1	1	1	4	3	−21.55	58.41	7.83
hist	0.5016	0.0239	0	0.0648	1	log_linear	1	1	1	3	4	−16.52	0.96	9.56
simple	0	0	1	0.0388	1	thq	1	1	1	4	2	−16.07	57.69	6.13
simple	0	0.0175	1	0.0586	1	thq	1	1	1	4	2	−16.05	58.41	5.94
simple	0	0.4841	1	0	0.9919	thq	5	1	1	4	5	−15.32	69.23	4.76
hist_amount	0.7693	1	1	0.1815	0.9834	thq	3	9	2	5	9	−11.00	79.81	1.83
simple	0	0.7829	1	0	0.0960	thq	2	1	1	7	4	−7.84	71.88	4.30
hist_amount	0.5649	1	1	0.1079	0.8284	thq	2	5	2	1	11	−5.77	79.09	0.90
simple	0	0.8756	1	0.0230	0.0249	thq	1	1	1	7	4	−5.66	72.60	3.90
simple	0	1	1	0.0150	0.0955	thq	1	1	1	5	3	−4.31	72.84	2.75
simple	0.0080	0.4509	0.8997	0	1	thq	1	1	1	4	4	−3.79	64.18	4.80
simple	0	0.6847	1	0.2721	0	thq	1	1	1	5	2	−3.06	71.63	3.03
simple	0	0.8897	1	0	0.1217	thq	1	1	1	5	2	−2.98	72.60	2.78
simple	0	0.9967	1	0	0.1295	thq	1	1	1	5	2	−1.98	72.84	2.72
hist_amount	0.5499	1	0.9980	0.1203	0.8135	thq	2	3	1	1	10	−1.29	78.85	0.68
simple	0	0.9372	1	0.0065	0.0953	thq	1	1	1	4	2	−0.71	72.84	2.23
hist_amount	0.3104	0	0.0604	0.0677	1	log_linear	1	1	1	3	1	−0.37	20.19	1.73
simple	0	0.9291	0.9753	0.0493	0	thq	1	1	1	4	1	−0.35	72.60	2.23
simple	0	1	1	0.0371	0.046	thq	1	1	1	4	1	0.43	72.84	2.20

**Table A3:** [IAP] Results for pruned and quantized model with single retraining epoch added.

Pruning		Quantization		*Quality Score*	Sparsity (%)	% of Original Size; Equation (15)
Method	Fraction	Method	Bits			
simple	0.3143	thq	4	−32.61	37.26	6.31
simple	0.3276	thq	4	−31.24	41.11	6.19
simple	0.3488	thq	4	−29.72	41.35	6.06
simple	0.3611	thq	4	−20.52	45.43	5.82
simple	0.4671	thq	4	−18.23	47.60	4.70
simple	0.4682	thq	4	−16.35	48.08	4.46
simple	0.4770	thq	4	−1.44	48.32	4.33

**Table A4:** [PM2.5] Results for pruned and quantized model.

Pruning		Quantization		*Quality Score*	Sparsity (%)	% of Original Size; Equation (15)
Method	Fraction	Method	Bits			
simple	0.0116	linear	10	−0.02	4.63	30.07
simple	0.0346	linear	31	−0.02	13.63	86.79
simple	0.0312	linear	15	−0.01	13.46	42.30
simple	0.0378	thq	31	−0.01	14.75	86.01
simple	0.0380	log_linear	21	−0.01	14.75	58.27
simple	0	thq	9	−0.01	0	28.13
simple	0.0422	linear	31	−0.01	15.91	85.08
simple	0.0405	linear	14	−0.01	14.95	38.52
hist_amount	0.0744	linear	8	0.01	7.38	23.17
simple	0.0204	linear	13	0.01	9.83	38.04
hist	0	log_linear	7	0.01	0	21.88
hist	0.0144	thq	13	0.02	10.56	36.94
simple	0.0294	linear	10	0.04	11.48	28.40
simple	0.0218	linear	8	0.07	9.95	23.30
simple	0.0216	linear	7	0.12	9.95	20.39
hist	0.0526	linear	11	0.23	14.66	29.47
hist	0.0667	linear	9	0.33	15.90	23.77
hist_amount	0.1135	thq	7	0.34	11.18	19.39
simple	0	linear	6	0.34	0	18.75
simple	0.0237	linear	6	0.34	10.03	17.42
hist	0.0924	linear	11	0.37	19.22	28.26
hist	0.1153	linear	16	0.46	19.62	40.36
hist	0.1121	log_linear	13	0.47	19.60	32.84
hist	0.1145	linear	8	0.52	19.62	20.18
simple	0.0596	linear	31	0.53	20.73	80.74
simple	0.0675	log_linear	17	0.55	21.33	43.08
simple	0.0652	linear	13	0.56	21.13	33.25
simple	0.0862	linear	19	0.56	26.30	45.14
simple	0.0863	log_linear	18	0.56	26.30	42.77
simple	0.0625	linear	10	0.61	20.91	25.83
simple	0.0874	log_linear	16	0.63	26.40	37.83
hist	0.2085	thq	9	0.65	22.65	21.43
hist	0.1185	linear	5	0.65	19.70	12.57
hist	0.2323	linear	14	0.73	22.91	32.91
hist	0.2074	linear	8	0.80	22.65	19.05
simple	0.0786	linear	7	0.83	24.93	17.00
simple	0.0879	linear	18	0.85	27.37	42.44
simple	0.0908	linear	18	0.99	28.50	41.95
simple	0.0811	linear	13	0.99	25.13	31.30

**Table A5:** [PM2.5] Results for individualized pruning and quantization.

Pruning Method	Pruning Fraction					Quantization Method	Quantization Bits					*Quality Score*	Sparsity (%)	% of Original Size; Equation (15)
	LSTM			Dense			LSTM			Dense				
	W	U	b	W	b		W	U	b	W	b			
simple	0	0	0	0	1	log_linear	21	27	31	31	24	0	0	77.22
simple	0	0	0	0.0384	0.9334	thq	23	15	13	15	31	0	0	56.94
hist	0	0	0.4037	0.1777	0.5768	thq	10	9	10	18	20	0.13	17.43	29.45
hist	0.0167	0	0.1239	0.1858	1	thq	15	21	6	10	11	0.13	15.58	54.29
hist	0	0.0197	0.5189	0	0.4907	thq	8	5	9	7	18	0.24	20.81	18.76
hist	0.1092	0.2250	0.3793	0.1891	0.7621	thq	14	7	7	10	26	0.39	23.15	24.44
hist	0.1013	0	0.7607	0.0542	1	thq	8	7	4	18	29	0.65	22.99	21.25
hist	0.1233	0.2404	0.5094	0.2301	1	thq	8	14	5	8	21	0.70	25.22	26.54
hist	0.1545	0.2266	1	0.5053	0.9034	thq	9	9	4	21	30	0.72	28.05	21.46
hist	0	0	0.6568	0.2933	0.8720	thq	8	13	4	10	29	0.78	21.10	33.05
hist	0.0277	021	0.7045	0.0977	0.9789	thq	5	9	4	11	18	0.99	22.78	21.08

**Table A6:** [PM2.5] Results for pruned and quantized model with single retraining epoch added.

Pruning		Quantization		*Quality Score*	Sparsity (%)	% of Original Size; Equation (15)
Method	Fraction	Method	Bits			
simple	0.0115	linear	15	−0.04	4.63	45.10
simple	0.0118	log_linear	13	−0.03	4.64	39.06
hist_amount	0.0734	linear	31	−0.03	7.37	89.84
hist_amount	0.0732	thq	19	−0.03	7.37	55.06
hist_amount	0.0503	thq	9	−0.03	1.45	27.55
hist_amount	0.0722	log_linear	12	−0.03	7.34	34.82
hist_amount	0.0767	linear	13	−0.03	7.48	37.50
hist_amount	0.0527	thq	9	−0.03	1.69	27.30
hist_amount	0.0831	linear	31	−0.02	9.43	88.91
hist_amount	0.0396	thq	9	−0.02	2.77	28.06
simple	0.0369	log_linear	22	−0.02	14.68	61.23
simple	0.0177	log_linear	19	−0.01	7.76	56.20
simple	0.0159	linear	9	−0.01	6.72	26.76
simple	0	log_linear	7	0.01	0.00	21.88
simple	0	linear	8	0.02	1.43	24.51
hist_amount	0.1189	log_linear	11	0.02	11.30	30.32
hist_amount	0.0080	linear	8	0.02	2.35	24.49
hist	0.0405	log_linear	21	0.05	14.25	57.29
hist_amount	0.1085	thq	8	0.12	4.57	22.42
hist	0.0523	thq	16	0.13	14.79	42.63
hist	0.0522	log_linear	18	0.14	14.94	47.66
hist_amount	0.0104	thq	7	0.17	0.92	21.86
hist	0.1171	log_linear	22	0.18	19.80	55.06
hist	0.1020	log_linear	21	0.20	19.47	53.36
hist_amount	0.0134	linear	7	0.20	5.55	21.03
hist	0.2323	thq	27	0.24	23.11	62.84
simple	0.0430	thq	15	0.25	17.79	41.04
hist	0.2298	log_linear	26	0.27	23.08	60.60
hist	0.2331	log_linear	26	0.27	23.19	60.25
hist	0.2140	log_linear	18	0.30	23.52	41.02
simple	0	linear	6	0.32	10.13	17.35
simple	0.0491	thq	10	0.37	18.18	26.91
simple	0.0838	linear	31	0.43	26.18	74.12
simple	0.0676	linear	9	0.43	22.41	22.63
hist_amount	0.0318	thq	5	0.53	1.83	15.61
simple	0.0911	linear	22	0.68	28.56	51.13
simple	0.0917	linear	27	0.69	28.58	62.66
simple	0.0779	linear	16	0.80	24.00	38.95
simple	0.0943	linear	26	0.87	28.78	59.73
simple	0.0953	linear	31	0.98	28.83	71.07

**Table A7:** [LHC] Results for model trained on continuous data with individualized pruning and quantization applied.

Tensor	Pruning		Quantization		Accuracy Drop (p.p.)	Sparsity (%)		% of Original Size; Equation (15)
	Method	Fraction	Method	Bits		Tensor	Model	
$W_{1}$	hist	0.3399	log_minmax	31	0.7813	31.93	18.16	20.73
$U_{1}$		0.9195		5		39.48		
$b_{1}$		0		3		0		
$W_{2}$		0.4597		22		26.43		
$U_{2}$		0.1232		16		16.36		
$b_{2}$		0.4078		7		40.63		
$W_{D}$		0.7452		13		15.63		
$b_{D}$		0.9012		31		0		
$W_{1}$	hist	0.3428	linear	31	0.3198	32.03	22.94	21.88
$U_{1}$		0.9289		4		39.52		
$b_{1}$		0.0007		4		10.55		
$W_{2}$		0.5587		20		29.76		
$U_{2}$		0.0572		17		12.18		
$b_{2}$		0.4647		7		40.63		
$W_{D}$		0.8108		13		15.63		
$b_{D}$		0.8357		31		0		

**Table A8:** [**LHC**] Results for model trained on bucketized data with individualized pruning and quantization applied.

Tensor	Pruning		Quantization		Accuracy Drop (p.p.)	Sparsity (%)		% of Original Size; Equation (15)
	Method	Fraction	Method	Bits		Tensor	Model	
$W_{1}$	simple	0.6777	linear	16	−0.6860	2.15	82.62	9.69
$U_{1}$		0.0060		28		0		
$b_{1}$		0		31		99.61		
$W_{2}$		0.9868		22		63.00		
$U_{2}$		0.1782		27		90.41		
$b_{2}$		0.2978		1		98.44		
$W_{D}$		0.9626		23		95.31		
$b_{D}$		0.2765		28		0		
$W_{1}$	simple	0.7577	linear	18	−0.6787	0	81.44	11.71
$U_{1}$		0		26		0		
$b_{1}$		0		29		99.61		
$W_{2}$		0.9601		24		57.53		
$U_{2}$		0.1576		30		91.14		
$b_{2}$		0.3075		1		95.31		
$W_{D}$		0.9011		23		96.88		
$b_{D}$		0.3396		25		0		
$W_{1}$	simple	0.6342	linear	19	−0.4858	0	80.64	10.40
$U_{1}$		0		27		0		
$b_{1}$		0		28		99.61		
$W_{2}$		1		26		72.47		
$U_{2}$		0.2178		31		82.57		
$b_{2}$		0.2371		1		86.72		
$W_{D}$		0.8318		22		95.31		
$b_{D}$		0.4590		21		0		
$W_{1}$	simple	0.7174	linear	21	−0.2026	18.85	77.68	8.96
$U_{1}$		0.0498		29		0		
$b_{1}$		0		28		86.72		
$W_{2}$		0.7847		21		68.87		
$U_{2}$		0.2014		30		88.28		
$b_{2}$		0.2786		1		98.44		
$W_{D}$		0.9675		22		96.88		
$b_{D}$		0.3493		21		0		
$W_{1}$	simple	0.5817	linear	27	0	31.93	73.84	14.33
$U_{1}$		0.0861		26		5.98		
$b_{1}$		0.0117		22		87.11		
$W_{2}$		0.7933		24		48.36		
$U_{2}$		0.1279		31		86.57		
$b_{2}$		0.2644		1		88.28		
$W_{D}$		0.8437		25		93.75		
$b_{D}$		0.4712		20		0		
$W_{1}$	simple	0.4912	linear	26	0.3418		73.59	14.14
$U_{1}$		0.0046		28		1.66		
$b_{1}$		0		26		0		
$W_{2}$		0.7674		15		83.98		
$U_{2}$		0.2006		31		68.66		
$b_{2}$		0.0967		1		44.58		
$W_{D}$		1		28		99.22		
$b_{D}$		0.1240		27		87.50		
$W_{1}$	hist	0.7914	thq	31	0.4004	0	51.74	35.14
$U_{1}$		0		31		17.44		
$b_{1}$		0.1983		10		71.48		
$W_{2}$		0.9911		31		6.18		
$U_{2}$		0.0115		30		45.43		
$b_{2}$		1		1		64.84		
$W_{D}$		1		22		17.19		
$b_{D}$		0.9685		1		0		
$W_{1}$	hist	0.8226	thq	31	0.5737	0	51.74	35.43
$U_{1}$		0		29		17.61		
$b_{1}$		0.2031		10		71.48		
$W_{2}$		0.9073		31		6.01		
$U_{2}$		0.0106		31		45.29		
$b_{2}$		0.9923		1		64.84		
$W_{D}$		1		20		17.19		
$b_{D}$		0.9956		1		0		
$W_{1}$	hist	0.8290	thq	31	0.6885	17.77	51.23	35.39
$U_{1}$		0.0652		29		18.06		
$b_{1}$		0.2188		10		71.48		
$W_{2}$		0.9167		30		0		
$U_{2}$		0		30		44.97		
$b_{2}$		0.9700		1		64.84		
$W_{D}$		1		21		17.19		
$b_{D}$		0.9788		1		0		
$W_{1}$	hist	1	log_linear	31	0.7007	0	48.41	30.85
$U_{1}$		0		31		10.40		
$b_{1}$		0.0634		3		69.53		
$W_{2}$		0.7504		26		15.26		
$U_{2}$		0.1147		31		33.79		
$b_{2}$		0.4999		1		55.47		
$W_{D}$		0.5476		19		17.19		
$b_{D}$		0.4978		1		0		
$W_{1}$	hist	0.8802	thq	28	0.8643	0	48.33	37.87
$U_{1}$		0		31		8.03		
$b_{1}$		0.0369		1		71.48		
$W_{2}$		0.8672		30		0		
$U_{2}$		0		30		26.93		
$b_{2}$		0.3251		1		61.72		
$W_{D}$		0.9057		18		17.19		
$b_{D}$		0.5734		1		0		
$W_{1}$	hist	1	thq	31	0.8643	0	46.89	39.88
$U_{1}$		0		31		0		
$b_{1}$		0		1		71.48		
$W_{2}$		1		31		0		
$U_{2}$		0		31		23.07		
$b_{2}$		0.2392		1		60.16		
$W_{D}$		0.7507		23		17.19		
$b_{D}$		0.5214		1		0		
